# Mutations in the transcriptional regulator MAB_2885 confer tedizolid and linezolid resistance through the MmpS-MmpL efflux pump MAB_2302-MAB_2303 in *Mycobacterium abscessus*

**DOI:** 10.1371/journal.ppat.1013190

**Published:** 2025-05-30

**Authors:** Huiyun Zhang, Shiyong Wang, Yu Zhang, Wenya Hua, Siran Lin, Xinchang Chen, Tao Xu, Jiazhen Chen, Wenhong Zhang

**Affiliations:** 1 Department of Infectious Diseases, Shanghai Key Laboratory of Infectious Diseases and Biosafety Emergency Response, National Medical Center for Infectious Diseases, Huashan Hospital, Shanghai Medical College, Fudan University, China; 2 Shanghai Sci-Tech Inno Center for Infection & Immunity, Shanghai, China; New Jersey Medical School, UNITED STATES OF AMERICA

## Abstract

*Mycobacterium abscessus* (*MAB*) is a clinically significant multidrug-resistant (MDR) pathogen, particularly implicated in pulmonary infections among cystic fibrosis (CF) patients. Tedizolid (TZD), an oxazolidinone-class antibacterial drug, has been recommended as an alternative treatment for *MAB*-infected patients who are intolerant to or whose isolate is resistant to first-line drugs including linezolid (LZD). To investigate the TZD resistance mechanisms in *MAB*, we isolated 23 TZD-resistant *MAB* mutants and performed whole-genome sequencing (WGS) to identify resistance-associated genes. Frequent mutations were identified in *MAB_2885*, encoding a putative TetR transcriptional regulator, and *MAB_2303*, encoding a putative mycobacterial membrane protein large (MmpL). Drug susceptibility testing confirmed that *MAB_2885* mutations contribute to both TZD and LZD resistance in *MAB*. RNA-seq analysis revealed that restoring wild-type *MAB_2885* in mutants downregulated the *MAB_2302-MAB_2303*. Electrophoretic mobility shift assay (EMSA) showed the MAB_2885 protein binds to its target sequence upstream of *MAB_2302-MAB_2303*, further confirming their regulatory relationship. The W91R mutation in the MAB_2885 protein was found to impair its DNA-binding activity compared to the wild-type. Liquid chromatography-tandem mass spectrometry (LC-MS/MS) analysis confirmed that MAB_2302-MAB_2303 functions as a TZD efflux pump. Additionally, overexpression of *MAB_2885* in *M. abscessus* subsp. *bolletii and M. abscessus* subsp. *massiliense* also increased their TZD susceptibility and downregulated their respective MmpS-MmpL orthologs. Overall, our study demonstrates that mutations in *MAB_ 2885* contribute to TZD and LZD resistance by disrupting the negative regulation of the downstream MAB_2302-MAB_2303, which functions as a direct efflux pump for TZD. These findings provide new insights into oxazolidinone resistance mechanisms in *MAB* and identify potential biomarkers for detecting drug resistance.

## Author summary

Oxazolidinones are critical for treating *Mycobacterium abscessus (MAB)* infections*,* which present significant challenges to current therapeutic strategies. While ribosomal mutations have been the primary known mechanism of oxazolidinone resistance, emerging evidence suggests that TetR regulator mutations may also contribute to resistance in *MAB*, although the underlying mechanisms remain unclear. Our study identified mutations in the TetR regulator MAB_2885 that confer resistance to tedizolid (TZD) and linezolid (LZD) by upregulating the MmpS-MmpL efflux pump system encoded by *MAB_2302-MAB_2303*. Using LC-MS/MS analysis, we demonstrated that MAB_2302-MAB_2303 functions as a direct TZD efflux pump. Furthermore, we confirmed that this resistance mechanism is conserved across *M. abscessus* subspecies, including *M. abscessus* subsp. *bolletii* and *M. abscessus* subsp. *massiliense*. These findings reveal a novel resistance mechanism involving the TetR regulator MAB_2885 and its regulation of the MmpS-MmpL efflux pump system in *MAB*, which has important implications for clinical drug susceptibility testing and treatment strategies.

## Introduction

*Mycobacterium abscessus* (*MAB*) is a rapidly growing mycobacteria (RGM) belonging to the *Mycobacterium chelonae-M. abscessus* group, which includes closely related species such as *M. chelonae*, *M. abscessus* subsp. *abscessus*, *M. abscessus* subsp. *massiliense*, and *M. abscessus* subsp. *bolletii* [1]. Among these, *MAB* is the most clinically significant multidrug-resistant (MDR) pathogen in this group, accounting for approximately 65%-80% of RGM-related lung infections [2,3], particularly in patients with chronic lung diseases such as cystic fibrosis (CF) or bronchiectasis. *MAB* has also been implicated in nosocomial infections, further highlighting its clinical importance [4,5]. The treatment of *MAB* infections remains challenging due to its intrinsic resistance to most antibiotics, with reported cure rates of only 30%-50% [6]. Current guidelines recommend combination therapy with multiple drugs, including linezolid (LZD), clarithromycin, amikacin, cefoxitin, imipenem, and tigecycline [7]. Typically, this regimen requires administration of 3–4 antibiotics for 2–4 months before clinical and microbiological improvements are observed [8].

Tedizolid (TZD), a novel oxazolidinone antibiotic structurally similar to LZD, has demonstrated greater antibacterial activity against clinical *MAB* strains compared to LZD, and has been recommended as an alternative treatment for *MAB* infections [9–11]. Oxazolidinones uniquely inhibit bacterial protein synthesis by binding to the *23S rRNA*, making cross-resistance with other anti-tuberculosis drugs uncommon. This property has led to their inclusion in regimens for multidrug-resistant tuberculosis (MDR-TB) [12].

In *Mycobacterium tuberculosis* (*MTB*), mutations in *23S rRNA* and ribosomal protein genes, such as *rrl* and *rplC*, have been identified as the primary mechanisms of LZD resistance [13,14]. Recently, mutations in the *mce3R*, encoding a TetR family transcriptional repressor, were found to confer resistance to contezolid, another oxazolidinone, in *MTB* [15]. In *MAB*, a previous study confirmed that high-level resistance to LZD and other oxazolidinones, including TZD, results from on-target ribosomal mutations, while low-level cross-resistance was attributed to mutations in the TetR regulator MAB_4384, which represses the MmpL5-MmpS5 efflux pump [16]. Notably, a recent study demonstrated that MAB_2303, a novel *M. abscessus*-specific MmpL protein, functions as an LZD efflux pump and identified several other MmpL and MmpS proteins potentially involved in LZD resistance [17].

To further investigate the mechanisms of TZD resistance and develop rapid resistance detection methods for *MAB*, we characterized 23 in vitro-derived TZD-resistant mutants of the ATCC 19977 strain. Our study identified novel mutations in two genes associated with TZD resistance: *MAB_2885*, encoding a TetR family regulator, and *MAB_2303*, encoding an MmpL family efflux pump protein.

## Result

### Screening of TZD-resistant mutants and mutations identified by WGS

The minimum inhibitory concentration (MIC) of TZD for the parent strain ATCC 19977 was 8 µg/ml on 7H10^OADC^ plates. To obtain the TZD-resistant isolates, approximately 2 × 10^7^ CFUs of *MAB* ATCC 19977 were spread on 7H10^OADC^ plates containing 16 µg/ml TZD. After 7 days of incubation, 23 mutants were obtained from the plates, indicating a mutation frequency of approximately 1 × 10^-6^. The MIC for all 23 mutants on 7H10^OADC^ agar was ≥ 32 µg/ml, at least a fourfold increase compared to the wild-type (WT) strain. However, due to the aqueous solubility limit of TZD in 7H10^OADC^ plates, with a maximum concentration of 16 µg/ml, precise MIC values beyond this threshold could not be determined.

WGS analysis revealed that 21 out of 23 (91%) isolates carried non-synonymous mutations in either *MAB_2885* (11 isolates) or *MAB_2303* (11 isolates), with one isolate (T10) harboring mutations in both genes (Table 1). Additionally, two isolates had mutations in *MAB_2302* and *rrl*, respectively. Among the 11 isolates with mutations in *MAB_2885*, which encodes a putative TetR family transcriptional regulator, five different non-synonymous SNVs and one insertion mutation were identified. For *MAB_2303*, which encodes an MmpL membrane protein, three different SNVs were found across 11 isolates. Meanwhile, 15 of the 21 isolates also carried mutations in other genes, including *MAB_2712c, MAB_4797, MAB_0409, MAB_4746, MAB_1695, MAB_1411, MAB_1906, MAB_3934c, MAB_4099c* and *MAB_4951c* (Table 1).

**Table 1:** Mutations identified in 23 tedizolid-resistant *MAB* mutants by whole-genome sequencing.

Strains	Mutation in *MAB_2885* (amino acid change)	Mutation in *MAB_2303* (amino acid change)	Other genes		
			Gene	Nucleotide mutation (Amino acid change)	Gene product
**T1**	G55A (A19T)	–	–	*–*	–
T2	G127A (D43N)	–	*MAB_3934c*	G388T (E130STOP)	Possible hydrolase alpha beta fold
T3	G127A (D43N)	–	*MAB_2712c*	G722T (R241L)	Probable methylmalonyl-CoA mutase small subunit MutA
T4,T5	G127A (D43N)	–	*MAB_2712c*	G722T (R241L)	
T6	G127A (D43N)	– –	*MAB_2712c*	G722T (R241L)	
			*MAB_4951c*	C55G (S185R)	Methyltransferase GidB (glucose-inhibited division protein B)
T22	G127A (D43N)	–	*MAB_4797*	C860T (T287I)	Hypothetical luciferase-like monooxygenase
			*MAB_2712c*	G722T (R241L)	Probable methylmalonyl-CoA mutase small subunit MutA
**T7**	T140C (V47A)	–	–	–	–
T8	A257C (H86P)	–	*MAB_1906*	C82A (P28T)	Pyridoxamine 5’-phosphate oxidase-related
T9	T271C (W91R)	–	*MAB_1411*	C531A (D177E)	Putative mechanosensitive ion channel
T10	ins4C (FSC2)	G775C (A838P)	*MAB_4099c*	C3611T (S1204F)	Probable non-ribosomal peptide synthetase
T11	–	T820A (S274T)	*MAB_0409*	ins-89C (5’ intergenic insertion)	Putative transcriptional regulator WhiB4
**T12,T13,T14,T15**	–	T820A (S274T)	–	–	–
T16,T17,T18,T19	–	C2512T (H838Y)	*MAB_4746*	ins2598G (FSC866)	Putative membrane protein MmpL
T20	–	C2512T (H838Y)	*MAB_1695*	del504C (FSC168)	Putative Mce family protein
**T21**	–	–	*MAB_2302*	TA348AG (5’ intergenic SNV)	Putative membrane protein MmpS
**T23**	–	–	*MAB_r5052(rrl)*	A2665T	23S ribosomal RNA

a Ins, insertion; Del, deletion; FSC, frame shift codon; STOP, stop codon; SNV, single nucleotide variation

b Bold style indicates the mutant with only one mutation

### Mutations in *MAB_2885* and overexpression of *MAB_2302-MAB_2303* confer TZD and LZD resistance in *MAB*

Given that mutations in *MAB_2885* and *MAB_2303* were the most frequent (91%, 21/23 isolates), we investigated their roles in conferring TZD resistance in the WT strain and five TZD-resistant mutants (T1, T3, T7, T8 and T9), each harboring distinct mutations in *MAB_2885*. The overexpression plasmids pMV261BL::*MAB_2302-MAB_2303* and pMV261BL::*MAB_2885* were transformed into *MAB* ATCC 19977, followed by drug susceptibility testing.

**Fig 1:**
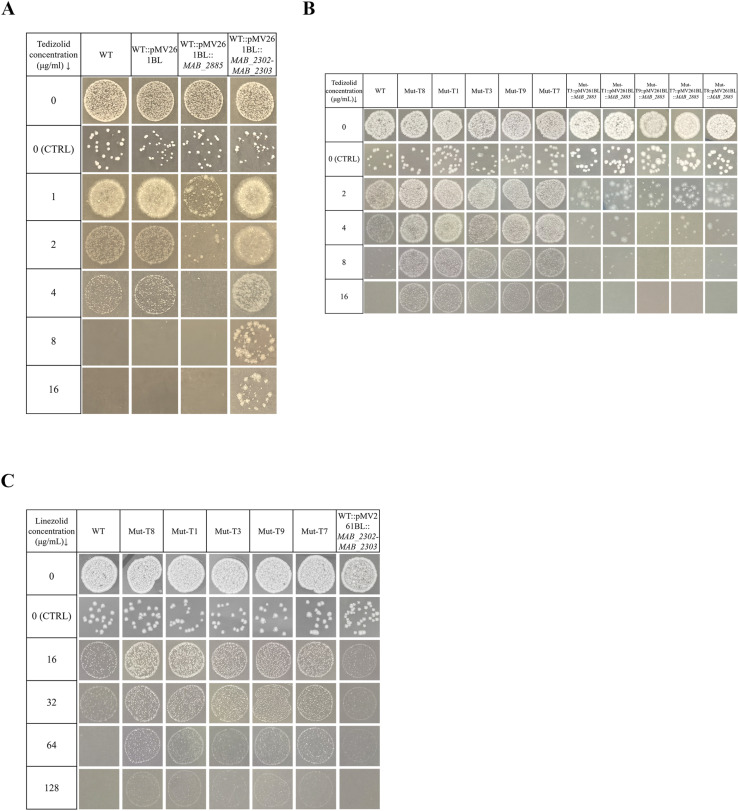
Oxazolidinone susceptibility testing of *MAB_2885* mutants, complemented strains, and *MAB_2302-MAB_2303* overexpression strain. (A) *MAB_2885* overexpression decreased TZD resistance, while *MAB_2302-MAB_2303* overexpression increased it compared to WT and empty plasmid control. (B) Complementation of *MAB_2885* in five mutants restored sensitivity to TZD. (C) LZD resistance was assessed in the five mutants with SNPs in *MAB_2885* and the isolate overexpressing the *MAB_2302-MAB_2303*. Approximately 2 × 10^3^ CFUs of bacterial suspension was spotted on 7H10^OADC^ agar with and without TZD, while about 20 CFUs bacteria were inoculated on 7H10^OADC^ plates without TZD as a control (CTRL).

As shown in Fig 1A, the empty plasmid pMV261BL showed no noticeable effect on TZD susceptibility in the WT strain (MIC = 8 µg/ml). Overexpression of *MAB_2885* reduced TZD resistance twofold (MIC = 4 µg/ml), while overexpression of *MAB_2302-MAB_2303* operon increased TZD resistance at least fourfold (MIC ≥ 32 µg/ml) (Fig 1A). Additionally, when *MAB_2885* was complemented, significant reductions in TZD resistance were observed in all five *MAB_2885* mutants with MICs returning to 8 µg/ml, the same as the WT strain (Fig 1B). We also evaluated LZD susceptibility in five mutants containing different SNPs in *MAB_2885* and in isolates overexpressing the *MAB_2302-MAB_2303* operon (Fig 1C). All five mutants showed LZD resistance, with MICs exceeding 128 µg/ml, representing at least a fourfold increase compared to the WT strain (MIC = 32 µg/ml). Overexpression of *MAB_2302-MAB_2303* operon resulted in a twofold increase in LZD resistance, with an MIC of 64 µg/ml. We also determined the MICs of TZD and LZD for the five *MAB_2885* mutants using the broth microdilution method (S1 Text). The MICs of TZD for all five mutants were 8 µg/ml, representing a fourfold increase compared to the WT strain (MIC = 2 µg/ml). Similarly, the LZD MICs for the mutants also showed a fourfold increase (MIC = 128 µg/ml) relative to the WT strain (MIC = 32 µg/ml) (S1 Table). Both approaches consistently demonstrated that the *MAB_2885* mutants displayed significantly enhanced resistance to both TZD and LZD compared to the WT strain. These findings suggest that dysfunction of *MAB_2885* and upregulation of the MmpS-MmpL efflux pump, encoded by *MAB_2302-MAB_2303*, contribute to the significantly increased resistance to TZD and LZD in *MAB*.

### *MAB_2885* negatively regulates expression of *MAB_2302-MAB_2303* efflux pump

**Fig 2:**
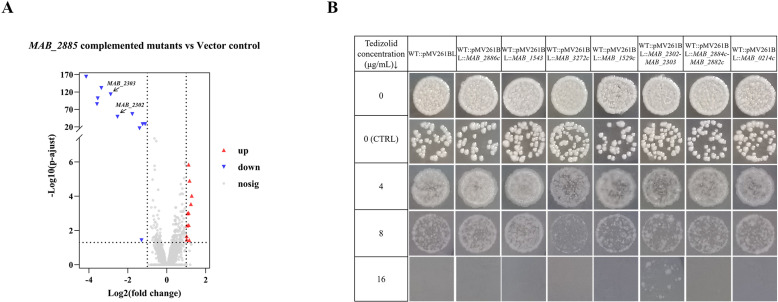
*MAB_2885* downregulates *MAB_2302-MAB_2303* operon, whose overexpression confers tedizolid resistance. (A) Volcano plots of differential expression of gene transcripts comparing the *MAB_2885* complemented group with the control group harboring empty plasmids. Log2FC <−1 or > 1, p-adjust < 0.05. The x-axis represents the log2 scale of the fold change of gene expression, with positive values indicating up-regulation and negative values indicating down-regulation. The y-axis shows the minus log10 scale of the adjusted p-values (-log10 (p-adjust)), representing the significance level of expression differences. (B) TZD susceptibility tests for the strains overexpressing the 7 candidate genes or operons. Only the *MAB_2302-MAB_2303* operon overexpression strain showed increased TZD resistance.

To investigate the mechanism of TZD resistance caused by dysfunction of *MAB_2885*, we performed RNA-seq analysis comparing the *MAB_2885* complemented group (both T1 and T7 with pMV261BL::*MAB_2885*) with the control group (T1 and T7 with the empty pMV261BL plasmid). We identified 11 significantly up-regulated and 11 down-regulated genes, with log2FC values ranging from -4.15 to 1.28 (Fig 2A). Notably, *MAB_2302 and MAB_2303* were down-regulated by 5.78 and 7.39 times, respectively (Fig 2A). The list of down-regulated genes is provided in Table 2, and up-regulated genes in S4 Table.

**Table 2:** The 11 down-regulated differential genes and their expression products identified by the RNA-seq analysis.

Gene	log2(FC)	P-adjust	Gene product
*MAB_2886c*	-4.15	1.18E-164	Hypothetical protein
*MAB_1543*	-3.59	8.27E-86	Hypothetical protein
*MAB_3272c*	-3.55	1.28E-102	Probable cutinase Cut1
*MAB_1529c*	-3.36	4.19E-132	Hypothetical protein
*MAB_2303*	-2.89	2.89E-114	Putative membrane protein MmpL
*MAB_2302*	-2.53	1.17E-49	Probable conserved membrane protein MmpS
*MAB_2884c*	-1.77	2.07E-57	Probable crossover junction endodeoxyribonuclease RuvC
*MAB_0214c*	-1.40	1.58E-16	Conserved hypothetical protein (OsmC-like)
*MAB_3073*	-1.30	3.69E-02	Hypothetical protein
*MAB_2883c*	-1.24	1.88E-28	Holliday junction DNA helicase RuvA
*MAB_2882c*	-1.11	1.65E-28	Holliday junction DNA helicase RuvB

To further explore the relationship between TZD resistance and potential downstream genes of *MAB_2885*, we constructed overexpression strains for the seven most significantly down-regulated genes or operons, including *MAB_2886c, MAB_1543, MAB_3272c, MAB_1529c, 2302-MAB_2303, MAB_2884c-MAB_2882c,* and *MAB_0214c*. We observed that only the *MAB_2302-MAB_2303* overexpression strain showed the highest MIC, exceeding 16 µg/ml, which was at least twofold greater than the control strain (Fig 2B). However, the susceptibility of other overexpressed strains, including *MAB_2886c, MAB_1543, MAB_3272c, MAB_1529c, MAB_2884c-MAB_2882c* and *MAB_0214c*, showed no significant difference compared to the control strains.

Overall, these results indicated that *MAB_2885* functions as an inhibitory transcriptional factor regulating the expression of *MAB_2302-MAB_2303*, the only operon whose overexpression is associated with TZD resistance.

### MAB_2885 protein binds to sequence upstream of *MAB_2302*

**Fig 3:**
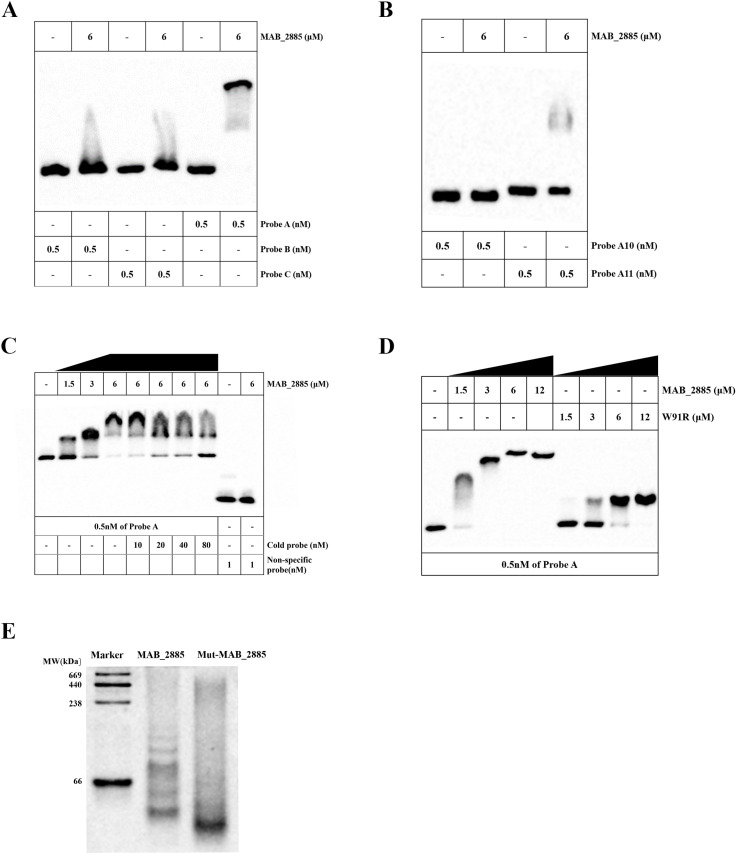
Binding activity of MAB_2885 protein to the intergenic region upstream of *MAB_2302-MAB_2303.* (A) EMSA using 5’ fluorescein-labeled probe A, B and C incubated with purified MAB_2885. (B) EMSA of two overlapping fragments derived from probe A. (C) Competition EMSA assay with probe A and purified MAB_2885 (D) EMSA comparing MAB_2885 W91R mutant and WT MAB_2885 at increasing concentrations with probe A. (E) Coomassie-blue-stained native-PAGE analysis of wild-type MAB_2885 and the MAB_2885 W91R mutant.

To identify the DNA binding site for MAB_2885, we performed EMSA experiments by incubating MAB_2885 protein with three DNA segments located upstream of *MAB_2302*, as detailed in the Methods section. Our results showed that the probe A specifically bound to MAB_2885 protein (Fig 3A). When we constructed two overlapping fragments containing the entire probe A: probe A10 (from -519 to -420 of *MAB_2302*) and probe A11 (from -430 to -321 of *MAB_2302*), we found that probe A10 did not bind to MAB_2885, while probe A11 exhibited very weak binding (Fig 3B). Since both probe A10 and probe A11 showed significantly weaker binding than probe A at the same concentration, this suggests that the complete probe A sequence is essential for effective binding to MAB_2885. Furthermore, we confirmed the specificity of probe A binding through a competition assay using increasing concentrations of MAB_2885 and an unlabeled DNA fragment (cold probe), while MAB_2885 failed to bind to the non-specific probe (Fig 3C).

The W91R variant showed significantly impaired DNA-protein complex formation compared to MAB_2885 at equivalent protein concentrations (Fig 3D). The impaired complex formation was accompanied by faster electrophoretic migration, suggesting conformational differences likely caused by disrupted polymerization. Further native-PAGE analysis revealed that MAB_2885 protein formed a polymeric structure, while the W91R mutant was probably in a monomeric state (< 66 kDa) (Fig 3E).

Overall, these findings demonstrate the binding ability and specificity of MAB_2885 to the upstream region of *MAB_2302* and *MAB_2303*.

### Overexpression of *MAB_2302-MAB_2303* and mutation in *MAB_2885* enhance TZD efflux in *MAB*

**Fig 4:**
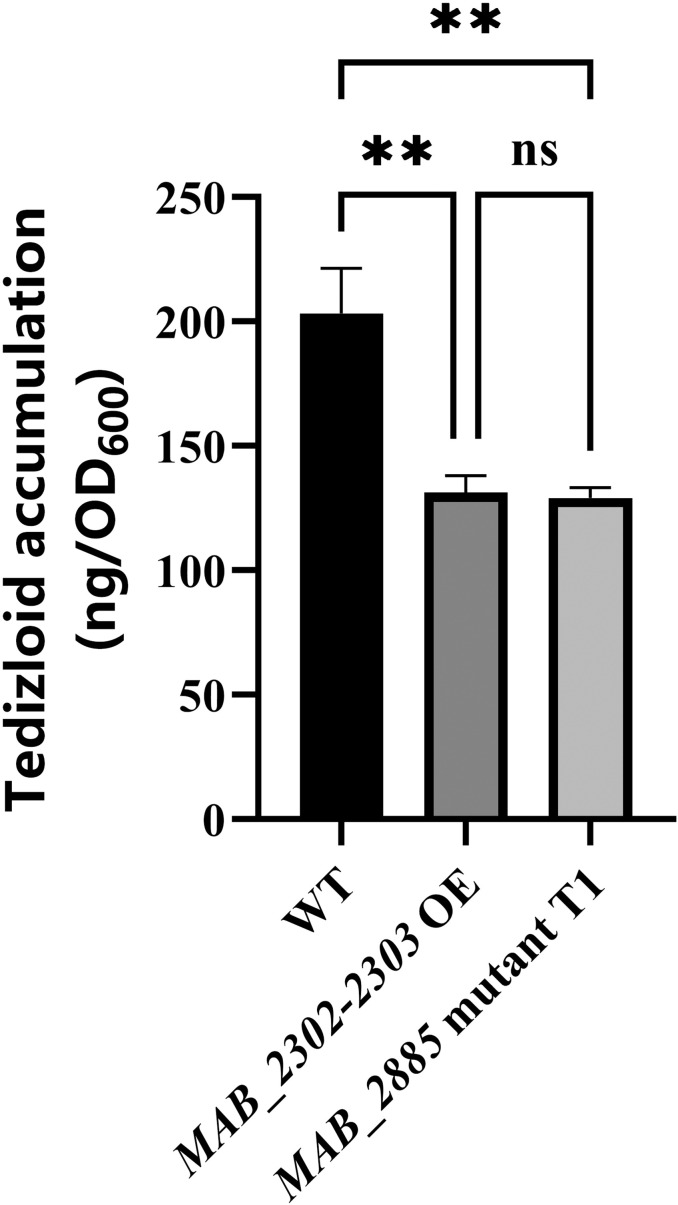
Intracellular TZD accumulation of the WT, *MAB_2885* mutant and *MAB_2302-MAB_2303* overexpression strain. TZD concentrations were measured by LC-MS/MS and normalized to OD600 to obtain accumulation levels. Data are represented as mean ± SEM from six biological replicates. Statistical significance was determined by one-way ANOVA. **p < 0.01, ns = non-significant, OE = overexpression strain.

In order to determine whether *MAB_2302-MAB_2303* and its regulator *MAB_2885* affect TZD accumulation, we quantified intracellular TZD concentrations using liquid chromatography-tandem mass spectrometry (LC-MS/MS). As shown in Fig 4, both the *MAB_2885* mutant strain T1 and *MAB_2302-MAB_2303* overexpression strain had significantly reduced intracellular TZD accumulation compared to the wild-type strain (p < 0.01), suggesting that MAB_2302- MAB_2303 functions as a TZD efflux pump.

### Conservation of *MAB_2885*-associated TZD resistance mechanisms across *M. chelonae-M. abscessus* group

**Fig 5:**
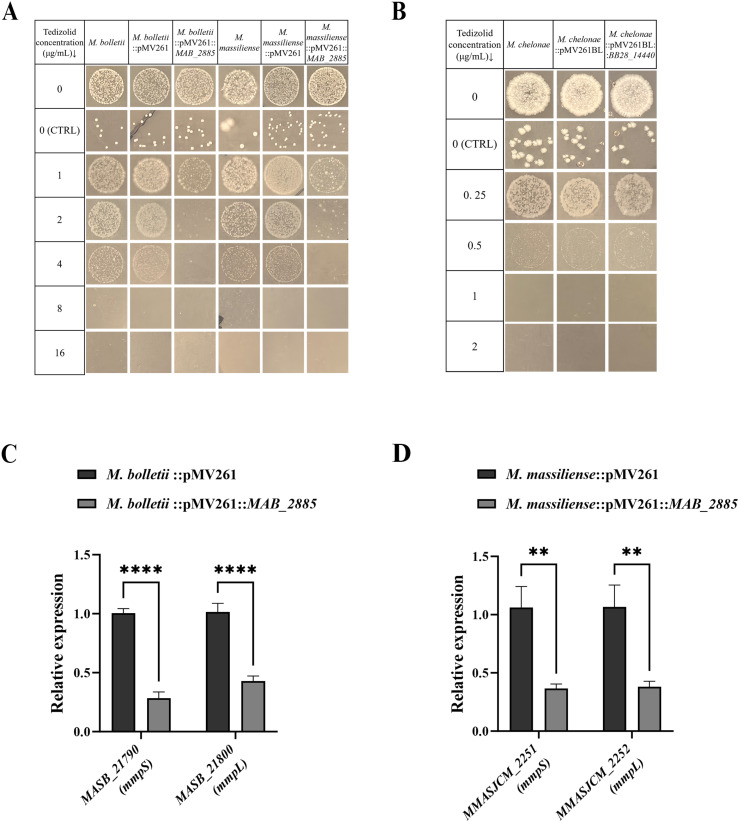
*MAB*_2885-mediated regulation of *mmpS-mmpL* and its impact on TZD susceptibility in the *M. abscessus* complex and *M. chelonae.* (A) Reduced TZD susceptibility in *M. abscessus* subsp. *bolletii* and *M. abscessus* subsp. *massiliense* overexpressing *MAB_2885* compared with the wild-type strains and the empty vector control. (B) Overexpression of *BB28_14440* in *M. chelonae* CCUG 47445 showed no effect on TZD susceptibility. (C-D) RT-qPCR analysis of *mmpS-mmpL* expression in *M. abscessus* subsp. *bolletii* and *M. abscessus* subsp. *massiliense* overexpressing *MAB_2885*, compared with the wild-type strain containing empty vector. Data are represented as mean ± SEM from five biological replicates. **, p < 0.01. ****, p < 0.0001.

To determine whether these resistance mechanisms are common among subspecies within the *M. chelonae-M. abscessus* group, we analyzed the closest orthologous amino acid sequences of the MAB_2885 protein. We found that MASB_RS13985 and MMASJCM_2844, from *M. abscessus* subsp. *bolletii* and M*. abscessus* subsp. *massiliense*, respectively, are identical to MAB_2885 in their amino acid sequence. In *M. chelonae*, the closest ortholog, BB28_14440, exhibits 67.76% identity with MAB_2885 at the amino acid level. After overexpressing *MAB_2885* or its orthologs in different subspecies, the MICs of TZD decreased twofold (MIC = 4 µg/ml) in both *M. abscessus* subsp. *bolletii* and *M. abscessus* subsp. *massiliense* (Fig 5A), while remaining unchanged in *M. chelonae* (MIC = 1 µg/ml) (Fig 5B).

The MAB_2302-MAB_2303 orthologs were identified as MASB_21790 (100% identity)-MASB_21800 (99.8% identity) in *M. abscessus* subsp. *bolletii*, and MMASJCM_2251 (100% identity)*-*MMASJCM_2252 (99.5% identity) in *M. abscessus* subsp. *massiliense*. To further investigate TetR’s regulatory effect on downstream MmpS-MmpL, we performed RT-qPCR to evaluate the expression of *mmpS-mmpL* in these two subspecies of *M. abscessus* complex. The result showed that overexpression of *MAB_2885* significantly decreased *mmpS-mmpL* expression in both subspecies. *MASB_21790* and *MASB_21800* had reductions of 0.28-fold and 0.43-fold (p < 0.0001), respectively (Fig 5C), while *MMASJCM_2251* and *MMASJCM_2252* exhibited reductions of 0.37-fold and 0.38-fold (p < 0.01), respectively (Fig 5D). Our findings suggest that *MAB_2885*-mediated negative regulation of MmpS-MmpL contributes to TZD susceptibility across all three subspecies of the *M. abscessus* complex.

## Discussion

*MAB* infections are challenging to treat due to their intrinsic resistance to the classical anti-tuberculous drugs and most available antibiotics [18,19]. Although LZD has been a common oral therapy for *MAB* infections, its clinical use has been limited by high cost and associated adverse effects [20]. Recently, the American Thoracic Society (ATS) recommended TZD as an alternative antibiotic for treating nontuberculous mycobacteria (NTM) infections [11]. Our findings highlight the importance of drug susceptibility testing before initiating *MAB* treatment, especially given the rising resistance to both TZD and LZD associated with mutations in *MAB_2885* and *MAB_2302-MAB_2303.* These results can facilitate the development of molecular diagnostic assays for TZD resistance using PCR-based methods, including quantitative or digital PCR, as well as targeted next-generation sequencing (tNGS). These molecular approaches can identify resistance-associated mutations in key genes such as *MAB_2885 (tetR), MAB_r5052 (rrl), MAB_2303 (mmpS), MAB_3820c (rplc), MAB_4384(tetR)*, whose association with TZD resistance is supported by our findings and previous research [11].

The efflux pump system plays a crucial role in antibiotic resistance in *MAB* [21]. TetR family members contain a conserved N-terminal helix-turn-helix (HTH) DNA-binding domain and a C-terminal ligand regulatory domain [22]. Several studies have shown that TetR regulators influence drug sensitivity in *MAB* by regulating efflux pumps, including mutations in the TetR repressor MAB_4384 that upregulate the MmpS5-MmpL5 and increase resistance to thiacetazone derivatives and several oxazolidinones [16,23,24]. Additionally, elevated transcription of MmpL9 has been observed in LZD-resistant *MAB* isolates [25]. Another TetR regulator MAB_2299c was found to regulate the expression of the *MAB_2300-MAB_2301* and *MAB_1135c-1134c* operons, which encode two pairs of MmpS-MmpL efflux pumps involved in the resistance to clofazimine (CFZ) and bedaquiline (BDQ). Furthermore, this study verified that *MAB_2299c* is unable to bind to IR_2302/03_ or regulate the expression of the *MAB_2302-MAB_2303* MmpS-MmpL [26,27].

Typically, the DNA-binding motifs recognized by the TetR regulators consist of palindromic sequences or inverted repeats. However, MEME analysis did not predict such binding sites in our study. Using the specific regulatory binding site of MAB_2299c for *MAB_2300-MAB_2301* as a negative control, we confirmed the specific regulation of *MAB_2302-MAB_2303* by MAB_2885, indicating these adjacent pairs of MmpS-MmpL proteins are regulated by two different TetR factors. While several genes were downregulated by *MAB_2885* except *MAB_2302-MAB_2303*, our complementation studies suggested that *MAB_2886c*, *MAB_1543*, *MAB_1529c* and *MAB_3073* (encoding hypothetical proteins of unknown function) do not directly contribute to TZD resistance. Although their knockout could theoretically affect TZD susceptibility, the lack of functional information makes such predictions uncertain. For *MAB_3272c* (encoding a Cut1-like cutinase), previous studies in *MTB* have established its role in cell wall synthesis and virulence [28], suggesting knockout may primarily impact bacterial growth. Similarly, the essential RuvABC complex (encoded by *MAB_2883c*, *MAB_2884c* and *MAB_2882c*) is essential for DNA repair [29], making complete knockout unfeasible.

**Fig 6:**
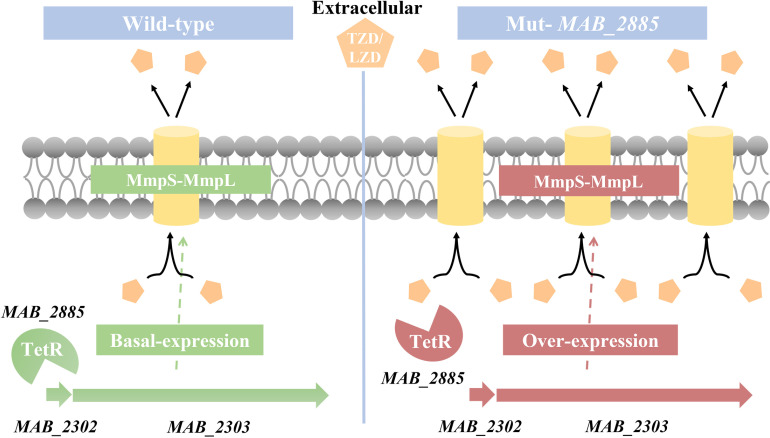
Model of TZD and LZD resistance mediated by *MAB_2885*-dependent MmpS-MmpL efflux pumps in *MAB.*

As shown in Fig 6, we speculated that *MAB_2302-MAB_2303* is negatively regulated by the TetR regulator encoded by *MAB_2885*. Mutations in *MAB_2885* weaken this repression, leading to increased efflux pump expression and resistance to TZD and LZD. Complementation with *MAB_2885* restores repression, downregulating the efflux pump and re-sensitizing resistant strains to both antibiotics.

However, our study still has several limitations. First, among the limited number of clinical *MAB* complex isolates in our previous study, only one isolate exhibited the highest MIC, with a 2-fold increase over wild-type [10], but no known mutation in *MAB_2885* was detected. Therefore, we are unable to assess the mutation frequency of *MAB_2885* in clinical TZD-resistant strains. Second, we could not precisely identify the binding sites of the MAB_2885 protein on the *MAB_2302-MAB_2303* operon. Presently, we can only localize these sites within a 180 bp region, which is relatively broad.

In conclusion, our study provides novel insights into the mechanistic roles of TetR-dependent regulation of the MmpS-MmpL efflux pump in *MAB* that confers oxazolidinones resistance, including TZD and LZD.

## Materials and methods

### Bacterial strains and culture conditions

The reference strains used in this study of the *M. chelonae-M. abscessus* group included *M. abscessus* subsp. *abscessu*s ATCC 19977 from the American Type Culture Collection (ATCC, USA), *M. abscessus* subsp. *bolletii* BD^T^ from the Collection of the Institut Pasteur (France), and M*. abscessus* subsp. *massiliense* CCUG 48898^T^ and *M. chelonae* CCUG 47445 from the Culture Collection of the University of Gothenburg (CCUG, Sweden). The parent strain and its derived strains are listed in S2 Table. All strains were cultured in 7H9 medium (BD Difco, USA) supplemented with 10% oleic acid, albumin, dextrose, and catalase (OADC) (7H9^OADC^) at 30°C.

### Screening of spontaneous TZD-resistant mutants and drug susceptibility testing

TZD and LZD were purchased from Aladdin (Shanghai, China), and dissolved in dimethyl sulfoxide (DMSO, Sigma, USA) to a stock concentration of 6 mg/ml and 30 mg/ml, respectively. To obtain spontaneous TZD-resistant *MAB* isolates, 100 µl of log-phase *MAB* ATCC 19977 (2 × 10^7^ CFUs) was spread on 7H10 agar (BD Difco, USA) supplemented with 10% OADC (7H10^OADC^) and 16 µg/ml of TZD, and cultured for 7 days at 30°C. Single colonies from the TZD-containing plates were isolated, and the minimum inhibitory concentrations (MICs) of TZD were determined using the agar dilution method. TZD-resistant colonies were grown to log phase in 7H9^OADC^ broth, adjusted to 0.5 McFarland standard, and 10 µl of 1:100 dilutions were inoculated onto 7H10^OADC^ agar with TZD concentrations of 0, 1, 2, 4, 8, and 16 µg/ml. Additionally, 10 µl of a 1:10^4^ dilution was inoculated onto 7H10^OADC^ agar as a control. Plates were incubated at 30°C for 4 days, with ATCC 19977 as the control, and the MICs were determined afterward.

### Whole-genome sequencing (WGS) and target identification

Genomic DNA extraction, library construction, and WGS of the 23 TZD resistant mutants were performed as described previously [30]. Genomic DNA was extracted using a manual magnetic bead-based method. Mutant isolates were cultured in 25 ml 7H9^OADC^ medium at 30°C for 4 days. After centrifugation, the pellet was resuspended in 300 µl of Tris-EDTA (TE) buffer. The suspension was mixed with 150 µl of 0.1 mm silica beads (Biospec, USA), 150 µl of S buffer (5 M guanidine isothiocyanate and β-mercaptoethanol), and 2 µl Y-30 (Sigma, USA) solution in a disruption tube. Bead beating was performed for 1 minute using the Mini-Beadbeater (Biospec, USA), followed by centrifugation at 13,000 rpm for 2 minutes. The supernatant was transferred to a new 1.5 ml microcentrifuge tube, and 4 µl of RNase (Thermo, USA) was added, followed by incubation for 10 minutes. After another centrifugation at 13000 rpm for 5 minutes, 200 µl of polyethylene glycol (PEG) buffer and 50 µl of XP magnetic beads were added to the supernatant. The mixture was incubated for 2 minutes, and the beads were washed twice with 80% ethanol after discarding the supernatant using a magnetic rack. After air-drying, the beads were resuspended in 53 µl TE buffer, mixed, and incubated for 1 minute. A final centrifugation at 13,000 rpm for 10 minutes resulted in the collection of 50 µl DNA for downstream applications.

Libraries were prepared using the Nextera XT Sample Prep Kit (Illumina, USA) and sequenced on the Illumina Miseq or Illumina HiSeq platform following the manufacturer’s protocol, ensuring at least 100-fold coverage. Clean reads were obtained through quality control processes including adapter trimming, low-quality filtering, alignment, variant calling, and validation from the raw sequencing data. Clean reads were then aligned to the *MAB* ATCC 19977 reference genome (GenBank NC_010397) using Bowtie2. Only paired reads with both ends aligned to the reference genome were considered for single nucleotide variant (SNV) and indel (insertion and deletion) analysis. SNVs and indels ranging from 1 to 5 bp were sorted and called at a minimum sequencing depth of 20 reads.

### Plasmid construction

The strains and plasmids used in this study are listed in S2 Table, and the primers are provided in S3 Table. Target genes were amplified from genomic DNA of the reference strains using specific primers. *E. coli* DH5α was the host for all plasmid constructions, cultured at 37°C in LB medium supplemented with 50 μg/ml bleomycin or 50 μg/ml kanamycin (Thermo, USA).

For *M. abscessus*, *MAB_2885* and other candidate genes were cloned into the pMV261BL shuttle vector, a non-integrating *Mycobacterium* shuttle vector derived from pMV261 that substitutes kanamycin with bleomycin resistance. For *M. chelonae*, *BB28_14440* (67.76% amino acid identical to MAB_2885) was cloned into the pMV261BL. For *M. bolletii* and *M. massiliense*, *MAB_2885* was directly cloned into the pMV261. Recombinant plasmids were verified by Sanger sequencing, electroporated into *Mycobacterium* competent cells, and plated on 7H10^OADC^ agar containing 50 µg/ml bleomycin or kanamycin, followed by incubation at 30°C for 5–7 days. The empty pMV261BL and pMV261 vectors served as controls.

### RNA extraction and RT-qPCR (Reverse Transcription Quantitative PCR)

For RT-qPCR and RNA-seq analyses, isolates were cultured in 25 ml of 7H9^OADC^ medium at 30°C for 4 days, with 3 or 5 biological replicates. Plasmid-bearing isolates were cultured in 7H9^OADC^ medium supplemented with kanamycin or bleomycin. Total RNA from the isolates was extracted using the Bacterial RNA Kit (Omega, USA), and quantified with a NanoDrop instrument (Thermo, USA). RNA integrity and quality were assessed using an Agilent 2100 Bioanalyzer (Agilent Technologies, USA). Total RNA from isolates was treated with DNase I, and then cDNAs were synthesized using the PrimeScript RT Master Mix (Takara, Japan). For relative abundance analysis of RNA transcripts, each isolate was analyzed with five replicates, using *sigA* as the endogenous control as in the previous study [31]. The RT-qPCR amplification reaction utilized the TB Green Premix Ex Taq II kit (Takara, Japan) on a LightCycler 480 Real-Time System (Roche, Switzerland). Gene expression fold changes were calculated using the 2–ΔΔCt method. This experiment was conducted with five biological replicates and two technical replicates. The primer sequences for RT-qPCR are provided in S3 Table.

### RNA sequencing and data analysis

To investigate the mechanism of TZD resistance caused by mutations in *MAB_2885*, two mutants (T1 and T7), each harboring a distinct SNP in *MAB_2885*, were selected for RNA-seq analysis. The analysis included two experimental groups: a test group of T1 and T7 mutants complemented with *MAB_2885*, and a control group of T1 and T7 transformed with the empty pMV261BL plasmid.

The isolates were cultured, and total RNA was extracted as previously described, with three biological replicates. Library construction and RNA sequencing of the isolates were performed as described previously [32]. Sequencing libraries were prepared with the Illumina TruSeq RNA Sample Preparation Kit and sequenced on the Illumina HiSeq platform. Clean reads were aligned to *MAB* ATCC_19977 reference genome by using Bowtie2. Gene expression levels were quantified as fragments per kilobase of transcript per million mapped reads (FPKM). Differentially expressed genes (DEGs) between the two groups were identified using the DESeq2 package in R, based on a statistical analysis of multi-conditional expression data matrices [33]. P-values were calculated for each gene and adjusted for multiple comparisons using the Benjamini-Hochberg (BH) method to control the false discovery rate (FDR). Criteria for identifying differentially expressed genes included a fold change of ≥ 2 (absolute log2FC ≥ 1) and an adjusted p-value (p-adjust) < 0.05.

### Expression and purification of MAB_2885 protein and W91R variant

The expression and purification of MAB_2885 and the W91R variant were performed as described in previous studies [27,23]. Briefly, *Escherichia coli* strain BL21 Rosetta (DE3) cells were transformed with the pET28a and pET30a constructs, containing the wild-type and the W91R-mutated *MAB_2885* gene respectively. Cultures were grown in LB medium supplemented with 300 µg/ml kanamycin and 34 µg/ml chloramphenicol, until OD600 reached 0.6. Protein expression was induced by adding 0.5 mM IPTG, followed by overnight incubation at 20°C. Cells were harvested by centrifugation (6,000 × g, 4°C for 30 min), resuspended in lysis buffer (50 mM Tris-HCl, 300 mM NaCl, 0.2 mM PMSF, 0.1% Triton X-100, pH 8.0), and lysed by sonication. The lysate was clarified by centrifugation (20,000 × g, 4°C for 45 min), and crude protein was purified by immobilized metal affinity chromatography (IMAC) using Ni-NTA Sepharose (GE Healthcare, USA). The purified protein was dialyzed into 50 mM Tris, 300 mM NaCl, 0.1% Sarkosyl, and 2 mM DTT (pH 8.0), concentrated with PEG 20000, filtered through a 0.45 μm membrane, aliquoted, and stored at -80°C for subsequent experiments.

Native polyacrylamide gel electrophoresis (Native-PAGE) of the MAB_2885 and W91R variant proteins was performed using samples diluted to 0.1 mg/ml with 1 × Phosphate-Buffered Saline (PBS, Gibco, USA). A 16 μl aliquot of each sample was mixed with 4 μl of 5 × native loading buffer (Wanshenghaotian, China). The mixtures were loaded onto a 12% native Tris-glycine gel and subjected to electrophoresis at 90 V for 90 minutes on ice in Tris-glycine buffer. The gel was then stained with Coomassie Blue for one hour and destained overnight in distilled water for optimal visualization, with imaging performed using an Azure Imaging Systems 600 imager (Azure Biosystems, USA).

### Electrophoretic Mobility Shift Assays (EMSA)

**Fig 7:**
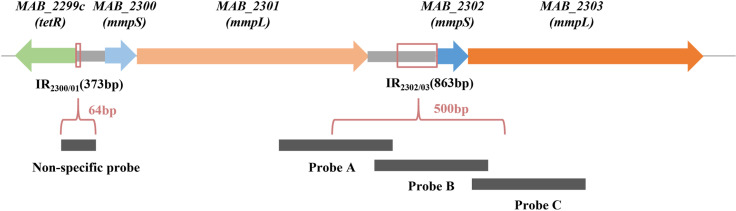
Division of the 500-bp upstream region of *MAB_2302* into three overlapping probes to explore the binding sites.

The Motif-based sequence analysis tool, MEME, was used to identify potential DNA binding motifs, as described in previous studies [24,27,34]. However, no typical palindromic sequences or inverted repeats were identified in the upstream 863-bp intergenic region of *MAB_2302*, consistent with a previous study [27]. Considering that the protein binding sites are usually located near the gene and the mutant T21 harbors a unique SNP (TA-348AG) in the 5’ intergenic region, we divided the 500-bp upstream region of *MAB_2302* into three overlapping probes to explore the binding sites (Fig 7), using the primers listed in the S3 Table. A non-specific probe, a 64-bp DNA segment between *MAB_2299c* and *MAB_2300*, served as a negative control and was previously shown to bind specifically to MAB_2299c [27].

The gel shift assay was conducted following the protocol by Hellman and Fried [35]. All probes were amplified from *M. abscessus* ATCC 19977 and labeled with biotin at their 5’ ends. The primers used are listed in S3 Table. Increasing amounts (1.5-12 μM) of purified MAB_2885 protein were co-incubated with 5 nM of the biotin-labeled probes in 0.5 × Tris-borate-EDTA (TBE) buffer for 1 h at room temperature. The samples were then loaded onto a 6% native polyacrylamide gel electrophoresed for 70 minutes at 100 V in 0.5 × TBE buffer. Gel shifts were visualized by biotin detection using an Azure Imaging Systems 600 imager.

### Intrabacterial TZD extraction

TZD extraction in *MAB* was performed following a protocol previously described for LZD [17]. Briefly, the wild-type strain ATCC 19977, mutant strain T1 and overexpression strain WT::pMV261BL::*MAB_2302-MAB_2303* were cultured to mid-log phase (OD600 = 0.6-0.8) in 7H9^OADC^ or 7H9^OADC^ supplemented with 50 ng/ml bleomycin. For each strain, six biological replicates and two blank controls, each with a volume of 50 ml, were prepared. Cultures were diluted to an OD600 of approximately 0.8, pelleted by centrifugation, and resuspended in 10 ml of fresh 7H9^OADC^. The OD600 was measured before the addition of TZD at a final concentration of 16 µg/ml, while an equivalent volume of DMSO was added to the blank controls. Cultures were incubated at 37°C with shaking at 150 rpm for 4 hours. After incubation, cultures were centrifuged and washed once with pre-chilled 1 × PBS.

For extraction, pellets were resuspended in pre-chilled 1ml of a 3:1:0.004 mixture of acetonitrile: methanol: formic acid. Bacterial lysis was performed using a bead beater with four cycles of 45 seconds each. Cell debris was removed by centrifugation, and 20 µl of the supernatant was transferred to a 96-well plate, mixed with 200 μl of internal standard solution containing 5ng/ml verapamil (Fisher Scientific, USA), vortexed for 5 minutes at 800 rpm, and centrifuged for 15 min at 4000 rpm at 4 °C. For analysis, 30 μl of supernatant was diluted with 30 μl of H_2_O, vortexed for 5 minutes, then directly injected for LC-MS/MS analysis.

### LC-MS/MS analysis of TZD

The LC-MS analysis of tedizolid was performed similarly to previous studies [17,36], using a SHIMADZU UPLC system equipped with SCIEX API 4000 mass spectrometer. Metabolite separation was achieved using an Agilent Poroshell 120 EC-C18 column (Sigma, USA) with a 3 µl injection volume. The mobile phase consisted of 0.1% formic acid in water (solvent A) and acetonitrile (solvent B), applied in the following gradient program: 0–0.80 min, linear gradient from 40% to 99%; 0.80–1.50 min, maintained at 90% B; 1.50–1.60 min, returned to 40% B; 1.60–2.00 min, equilibrated at 40% B. The flow rate was maintained at 1 ml/min, and the column temperature was set to 40°C.

The mass spectrometer was operated in positive ion mode with electrospray ionization (ESI), utilizing multiple reaction monitoring (MRM) for data acquisition. For TZD, the precursor ion [M + H]^+^ (m/z 371.3) was selected, and the characteristic product ion m/z 343.2 was monitored for quantification. Similarly, verapamil was monitored using its precursor ion [M + H]⁺ (m/z 455.23) and the product ion m/z 165.1. Following LC-MS analysis, TZD identification was conducted using Analyst 1.7 software (AB Sciex). Signal intensity was quantified based on a standard curve generated from TZD standards spiked in blank control cell lysates. TZD concentrations were determined from peak area ratios relative to the internal standard verapamil, with final accumulation values calculated after OD600 normalization.

### Data visualization and statistical analyses

Graphs and heatmaps were generated using GraphPad Prism software (version 10), and raw data are available in S1 Data. All quantitative data are reported as means ± SEM from at least three independent experiments. Intergroup comparisons were performed using one-way or two-way ANOVA, followed by Tukey’s or Sidak post-hoc test, respectively. A p-value of less than 0.05 was considered statistically significant. Asterisks indicate statistically significant differences in post hoc test comparisons (****, P < 0.0001; ***, P < 0.001; **, P < 0.05; *, P < 0.05; ns, non-significant).

## Supporting information

S1 Text Supplementary materials and methods. (DOCX)

S1 Table The MICs of TZD and LZD for WT and *MAB_2885* mutants determined by broth microdilution. (DOCX)

S2 Table Bacterial strains and plasmids constructed in this study. (DOCX)

S3 Table Primers used in this study. (DOCX)

S4 Table The 11 up-regulated differential genes and their expression products identified by the RNA-seq analysis. (DOCX)

S1 Data Raw values for plots displayed in this manuscript. (XLSX)

S2 Data Complete, uncropped EMSA and Complete, uncropped agar. (ZIP)
