# Supplementary material for: Mutations in the transcriptional regulator MAB_2885 confer tedizolid and linezolid resistance through the MmpS-MmpL efflux pump MAB_2302-MAB_2303 in Mycobacterium abscessus
Source: PLoS Pathog. 2025 May 30;21(5):e1013190. doi: 10.1371/journal.ppat.1013190 (PMC12136459; doi:10.1371/journal.ppat.1013190)
Supplement: S1 Text — (DOCX) [file ppat.1013190.s001.docx]

**Text S1. Supplementary materials and methods**

**Drug susceptibility testing by** **broth microdilution**

The broth microdilution method for drug susceptibility testing of *MAB* was performed in 96-well plates. The WT strain ATCC 19977 and five mutants (T1, T3, T7, T8, T9) were grown to log phase in 7H9^OADC^, and then adjusted to a 0.5 McFarland standard. A 50 µl aliquot of a 1:100 dilution of each culture was inoculated into 96-well plates containing 50 µl of 7H9^OADC^ with TZD concentrations ranging from 1 to 32 µg/ml. The MIC was defined as the concentration of antibiotic at which there was no visible growth. MICs were determined after incubating at 30 °C for 4 days, depending on the growth of the positive control wells containing no antibiotic.
