# Supplementary material for: Mutations in the transcriptional regulator MAB_2885 confer tedizolid and linezolid resistance through the MmpS-MmpL efflux pump MAB_2302-MAB_2303 in Mycobacterium abscessus
Source: PLoS Pathog. 2025 May 30;21(5):e1013190. doi: 10.1371/journal.ppat.1013190 (PMC12136459; doi:10.1371/journal.ppat.1013190)
Supplement: S1 Table — (DOCX) [file ppat.1013190.s002.docx]

**Table S1.** **The MICs of TZD and LZD for WT and *MAB_2885* mutants determined by broth microdilution**

| Strains | MIC of TZD (µg/ml) by broth microdilution | MIC of LZD (µg/ml) by broth microdilution | Mutation in *MAB_2885* |
| --- | --- | --- | --- |
| WT | 2 | 32 | - |
| T1 | 8 | 128 | G55A (A19T) |
| T3 | 8 | 128 | G127A (D43N) |
| T7 | 8 | 128 | T140C (V47A) |
| T8 | 8 | 128 | A257C (H86P) |
| T9 | 8 | 128 | T271C (W91R) |
