# Supplementary material for: Mutations in the transcriptional regulator MAB_2885 confer tedizolid and linezolid resistance through the MmpS-MmpL efflux pump MAB_2302-MAB_2303 in Mycobacterium abscessus
Source: PLoS Pathog. 2025 May 30;21(5):e1013190. doi: 10.1371/journal.ppat.1013190 (PMC12136459; doi:10.1371/journal.ppat.1013190)
Supplement: S2 Table — (DOCX) [file ppat.1013190.s003.docx]

**Table S2****. Bacterial strains and plasmids constructed in this study.**

| Strains or plasmids | Relevant characteristics | Origins |
| --- | --- | --- |
| DH5α | A cloning host of *E. coli* | Lab stock |
| BL21(DE3) | An expression host of *E. coli* | Lab stock |
| *M. abscessus* subsp*. abscessu*s ATCC 19977 | reference strain | Lab stock |
| *M. abscessus* subsp*. bolletii* BD^T^ | reference strain | Lab stock |
| M*. abscessus* subsp*. massiliense* CCUG 48898^T^ | reference strain | Lab stock |
| *M. chelonae* CCUG 47445 | reference strain | Lab stock |
| WT::pMV261BL | ATCC 19977 carrying pMV261BL | This work |
| WT::pMV261BL::*MAB_2885* | ATCC 19977 carrying pMV261BL::*MAB_2885* | This work |
| WT::pMV261BL::*MAB_2302-MAB_2303* | ATCC 19977 carrying pMV261BL::*MAB_2302-MAB_2303* | This work |
| WT::pMV261BL::*MAB_2886c* | ATCC 19977 carrying pMV261BL::*MAB_2886c* | This work |
| WT::pMV261BL::*MAB_1543* | ATCC 19977 carrying pMV261BL::*MAB_1543* | This work |
| WT::pMV261BL::*MAB_3272c* | ATCC 19977 carrying pMV261BL::*MAB_3272c* | This work |
| WT::pMV261BL::*MAB_1529c* | ATCC 19977 carrying pMV261BL::*MAB_1529c* | This work |
| WT::pMV261BL::*MAB_2884c-MAB_2882c* | ATCC 19977 carrying pMV261BL::*MAB_2884c-MAB_2882c* | This work |
| WT::pMV261BL::*MAB_0214c* | ATCC 19977 carrying pMV261BL::*MAB_0214c* | This work |
| Mut-T1::pMV261BL | Mut-T1 carrying pMV261BL | This work |
| Mut-T3::pMV261BL | Mut-T3 carrying pMV261BL | This work |
| Mut-T7::pMV261BL | Mut-T7 carrying pMV261BL | This work |
| Mut-T8::pMV261BL | Mut-T8 carrying pMV261BL | This work |
| Mut-T9::pMV261BL | Mut-T9 carrying pMV261BL | This work |
| Mut-T1::pMV261BL::*MAB_2885* | Mut-T1 carrying pMV261BL::*MAB_2885* | This work |
| Mut-T3::pMV261BL::MAB_2885 | Mut-T3 carrying pMV261BL::*MAB_2885* | This work |
| Mut-T7::pMV261BL::*MAB_2885* | Mut-T7 carrying pMV261BL::*MAB_2885* | This work |
| Mut-T8::pMV261BL::*MAB_2885* | Mut-T8 carrying pMV261BL::*MAB_2885* | This work |
| Mut-T9::pMV261BL::*MAB_2885* | Mut-T9 carrying pMV261BL::*MAB_2885* | This work |
| *M. bolletii*::pMV261 | *M. abscessus* subsp*. bolletii* BD^T^ carrying pMV261 | This work |
| *M. bolletii*::pMV261::*MAB_2885* | *M. abscessus* subsp*. bolletii* BD^T^ carrying pMV261::*MAB_2885* | This work |
| *M. massiliense*::pMV261 | M*. abscessus* subsp*. massiliense* CCUG 48898^T^ carrying pMV261 | This work |
| *M. massiliense*:: pMV261::*MAB_2885* | M*. abscessus* subsp*. massiliense* CCUG 48898^T^  carrying pMV261::*MAB_2885* | This work |
| *M. chelonae*::pMV261BL | *M. chelonae* CCUG 47445 carrying pMV261BL | This work |
| *M. chelonae*:: pMV261BL::*BB28_14440* | *M. chelonae* CCUG 47445 carrying pMV261BL::*BB28_14440* | This work |
| Plasmid |  |  |
| pMV261 | A shuttle vector between *E. coli* and *Mycobacteria*, Km^R^ | Lab stock [1] |
| pMV261BL | pMV261 derivative, Ble^R^ | This work |
| pET28a | A T7 promoter expression vector; Km^R^ | Lab stock |
| pET30a | A T7 promoter expression vector; Km^R^ | Lab stock |
| pMV261BL::*MAB_2885* | pMV261BL carrying *MAB_2885* | This work |
| pMV261BL::*MAB_2302-MAB_2303* | pMV261BL carrying *MAB_2302-MAB_2303* | This work |
| pMV261BL::*MAB_2886c* | pMV261BL carrying *MAB_2886c* | This work |
| pMV261BL::*MAB_1543* | pMV261BL carrying *MAB_1543* | This work |
| pMV261BL::*MAB_3272c* | pMV261BL carrying *MAB_3272c* | This work |
| pMV261BL::*MAB_1529c* | pMV261BL carrying *MAB_1529c* | This work |
| pMV261BL::*MAB_2884c-MAB_2882c* | pMV261BL carrying *MAB_2884c-MAB_2882c* | This work |
| pMV261BL::*MAB_0214c* | pMV261BL carrying *MAB_0214c* | This work |
| pMV261BL::*BB28_14440* | pMV261BL carrying *BB28_14440* | This work |
| pMV261::MAB_2885 | pMV261 carrying MAB_2885 | This work |

**References**

1. Junqueira-Kipnis AP, de Oliveira FM, Trentini MM, Tiwari S, Chen B, Resende DP, et al. Prime-boost with Mycobacterium smegmatis recombinant vaccine improves protection in mice infected with Mycobacterium tuberculosis. PLoS One. 2013;8(11):e78639. pmid:24250805.
