# Supplementary material for: Mutations in the transcriptional regulator MAB_2885 confer tedizolid and linezolid resistance through the MmpS-MmpL efflux pump MAB_2302-MAB_2303 in Mycobacterium abscessus
Source: PLoS Pathog. 2025 May 30;21(5):e1013190. doi: 10.1371/journal.ppat.1013190 (PMC12136459; doi:10.1371/journal.ppat.1013190)
Supplement: S3 Table — (DOCX) [file ppat.1013190.s004.docx]

**Table S3.** **Primers used in this study**

| Primers | 5’to 3’sequence |
| --- | --- |
| Cloning in pMV261BL and pMV261 | |
| MAB_2885-F_PstⅠ | TGAACTGCAGATGCCCCCTGAACCTCGGCG |
| MAB_2885-R_Hind Ⅲ | ACTGAAGCTTCTAGGGCGCAGGGGTGTCGATG |
| MAB_2302-2303-F_PstⅠ | TGAACTGCAGGTGGTGTTGGTGGTCGCGGC |
| MAB_2302-2303-R_Hind Ⅲ | ACTGAAGCTTTTAGACCGGCGGGCGGGG |
| MAB_2886c-F_PstⅠ | TGAACTGCAGTGTATGTCAGGCAGGCGTACA |
| MAB_2886c-R_Hind Ⅲ | ACTGAAGCTTCAGCAGTACATCGACACCCCT |
| MAB_1543-F_PstⅠ | TGAACTGCAGCATGAGGTCAACGGATGTAGA |
| MAB_1543-R_Hind Ⅲ | ACTGAAGCTTCTCAGGTACTCGACGTTCTTA |
| MAB_3272c-F_PstⅠ | TGAACTGCAGCCCTAATTAGTTCGCCCGTAG |
| MAB_3272c-R_Hind Ⅲ | ACTGAAGCTTGTACCAGGCGCATCCGAT |
| MAB_1529c-F_PstⅠ | TGAACTGCAGGCACGTTTACATTAGGCCCAT |
| MAB_1529c-R_Hind Ⅲ | ACTGAAGCTTTTTGGGAACGCGCGGATTGAT |
| MAB_2884-82c-F_BamHI | TGAAGGATCCGTGCGGTCTGTCCATGGTCGA |
| MAB_2884-82c-R_Hind Ⅲ | ACTGAAGCTTGGCCCTAGTGTCGCGTCAATC |
| MAB_0214c-F_PstⅠ | TGAACTGCAGTATTCATCACGGCTGCAACTG |
| MAB_0214c-R_Hind Ⅲ | ACTGAAGCTTGTACGGTGCTTTTTCCTTTGC |
| BB28_14440-F_PstⅠ | TGAACTGCAGAGCGCATGTCTACCGAACCTC |
| BB28_14440- R_ Hind Ⅲ | ACTGAAGCTTGCCCGTGGACGCAGCCCGAGG |
| Primer for RT-PCR |  |
| sigA_qF | CACATGGTCGAGGTCATCAA |
| sigA_qR | TGGATCTCCAGCACCTTCTC |
| MM-mmps_qF | TTCGGCTCCACCAACATCAA |
| MM-mmps_qR | CCAGATAGTTCACCTCGCCC |
| MM-mmpl_qF | ACACCTTCTACCGCGAACTG |
| MM-mmpl_qR | CTTGGTCCCCACGGAGATTC |
| MB-mmps_qF | TCTATCGGCTCCACGGGGTA |
| MB-mmps_qF | GCGACGACATTGGCAAAAACAGAA |
| MB-mmpl_qF | TAAAGAGCACTCGGTCTCGC |
| MB-mmpl_qF | AACCATGACCATGCTGTCGT |
| Probes used for EMSA | |
| Probe A_Fw | ATTAGCGATGGCAAACACCCT |
| Probe A_Rv | GCCCTAAGCACCCGCAATAGC |
| Probe B_Fw | CGGGTGCTTAGGGCGGTGAAC |
| Probe B_Rv | CGTGTTGTTTTTGCGGAGTTA |
| Probe C_Fw | CTGACTAACTCCGCAAAAACAA |
| Probe C_Rv | GACTAGTGGGATCCATACCCGC |
| Non-specific probe_Fw | GTGCGTAACGCACGCTACCGGGTGGTTCGTTGGCTCGCAAATCACACGCCGTGCGTTATCCTCG |
| Non-specific probe_Rv | CACGCATTGCGTGCGATGGCCCACCAAGCAACCGAGCGTTTAGTGTGCGGCACGCAATAGGAGC |
| Probe A10_Fw | CGGCATCGCACCATTGAGAAT |
| Probe A10_Rv | GCCTGCTTGCACATCGACATT |
| Probe A11_Fw | GTGCAAGCAGGCGCTTTCGTT |
| Probe A11_Rv | GCCCTAAGCACCCGCAATAGC |
