# Supplementary material for: Mutations in the transcriptional regulator MAB_2885 confer tedizolid and linezolid resistance through the MmpS-MmpL efflux pump MAB_2302-MAB_2303 in Mycobacterium abscessus
Source: PLoS Pathog. 2025 May 30;21(5):e1013190. doi: 10.1371/journal.ppat.1013190 (PMC12136459; doi:10.1371/journal.ppat.1013190)
Supplement: S4 Table — (DOCX) [file ppat.1013190.s005.docx]

**Table S4.** **The 11 up-regulated differential genes and their expression products identified by the RNA-seq analysis**

| Gene | | log2(FC) | P-adjust | Gene product |
| --- | --- | --- | --- | --- |
| *MAB_1628c* | 1.28 | | 9.49E-05 | Hypothetical protein |
| *MAB_0438c* | 1.24 | | 2.89E-04 | Hypothetical protein |
| *MAB_1704c* | 1.17 | | 1.27E-05 | 1-aminocyclopropane-1-carboxylate deaminase |
| *MAB_2607* | 1.15 | | 3.69E-02 | Putative flavin-containing monooxygenase |
| *MAB_3015* | 1.13 | | 4.88E-03 | Hypothetical protein |
| *MAB_1727c* | 1.12 | | 9.42E-04 | Bacteriophage protein |
| *MAB_3999* | 1.12 | | 1.45E-06 | Hypothetical protein |
| *MAB_2048c* | 1.07 | | 1.02E-03 | Probable cytochrome P450 |
| *MAB_3716* | 1.04 | | 5.16E-03 | Hypothetical protein |
| *MAB_3132c* | 1.02 | | 2.15E-02 | Hypothetical protein |
| *MAB_2142* | 1.02 | | 3.40E-02 | NADH-quinone oxidoreductase, I subunit NuoI |
