# Supplementary figures and images for: Mutations in the transcriptional regulator MAB_2885 confer tedizolid and linezolid resistance through the MmpS-MmpL efflux pump MAB_2302-MAB_2303 in Mycobacterium abscessus
Source: PLoS Pathog. 2025 May 30;21(5):e1013190. doi: 10.1371/journal.ppat.1013190 (PMC12136459; doi:10.1371/journal.ppat.1013190)

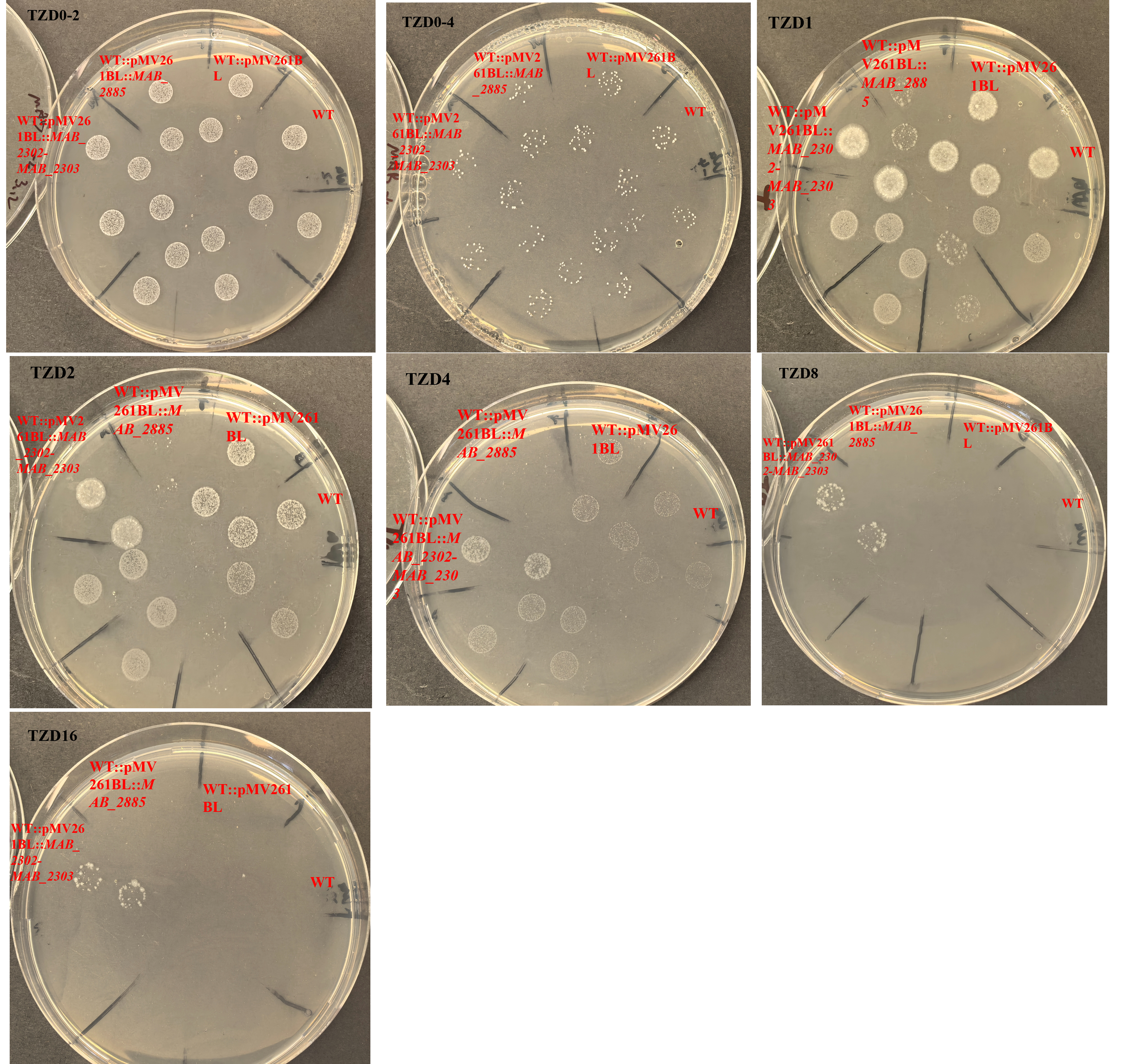

Supplement: S2 Data — (ZIP) [file ppat.1013190.s007.zip › S2_data/Complete, uncropped agar/Fig 1A.tif]

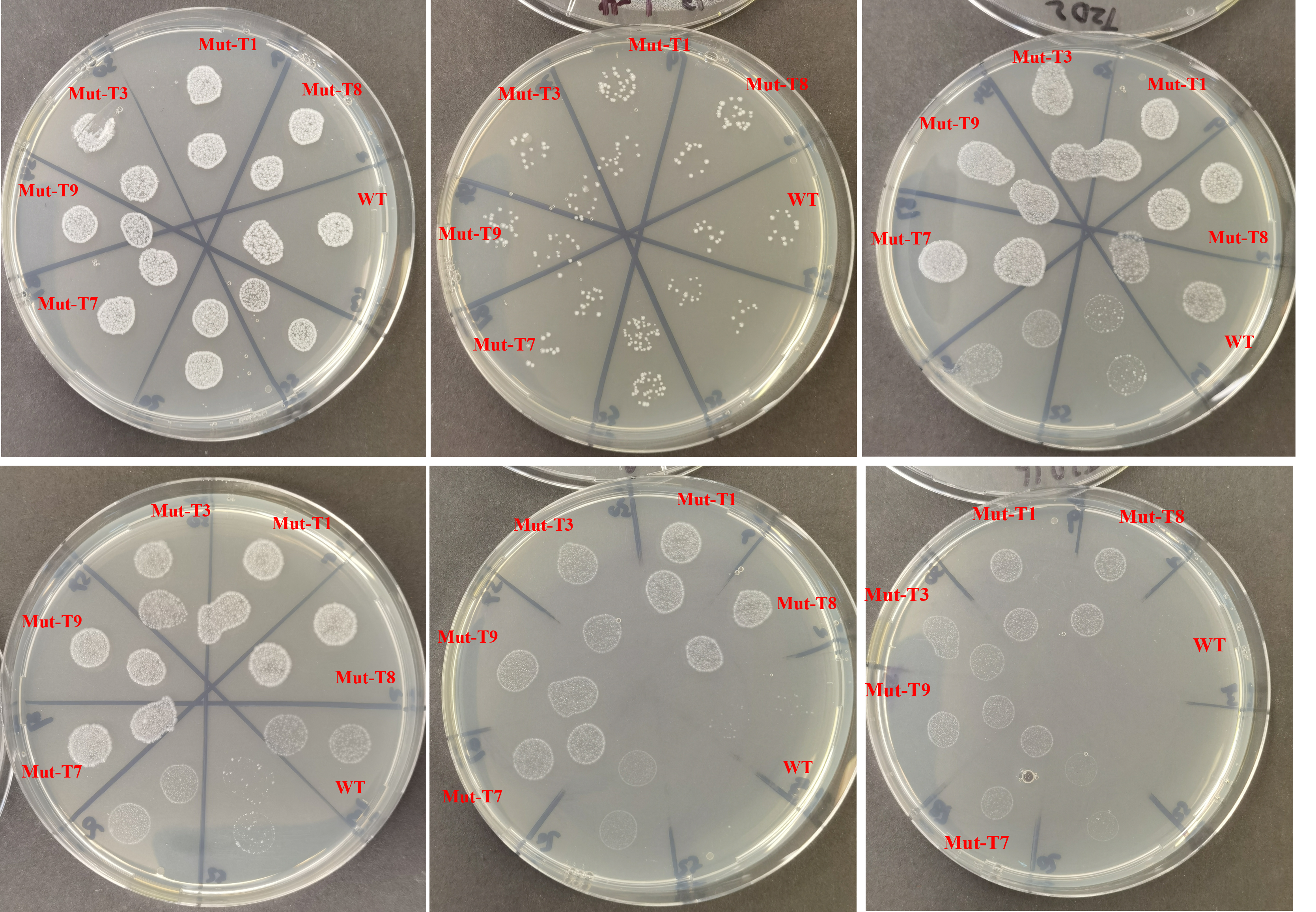

Supplement: S2 Data — (ZIP) [file ppat.1013190.s007.zip › S2_data/Complete, uncropped agar/Fig 1B-mut.tif]

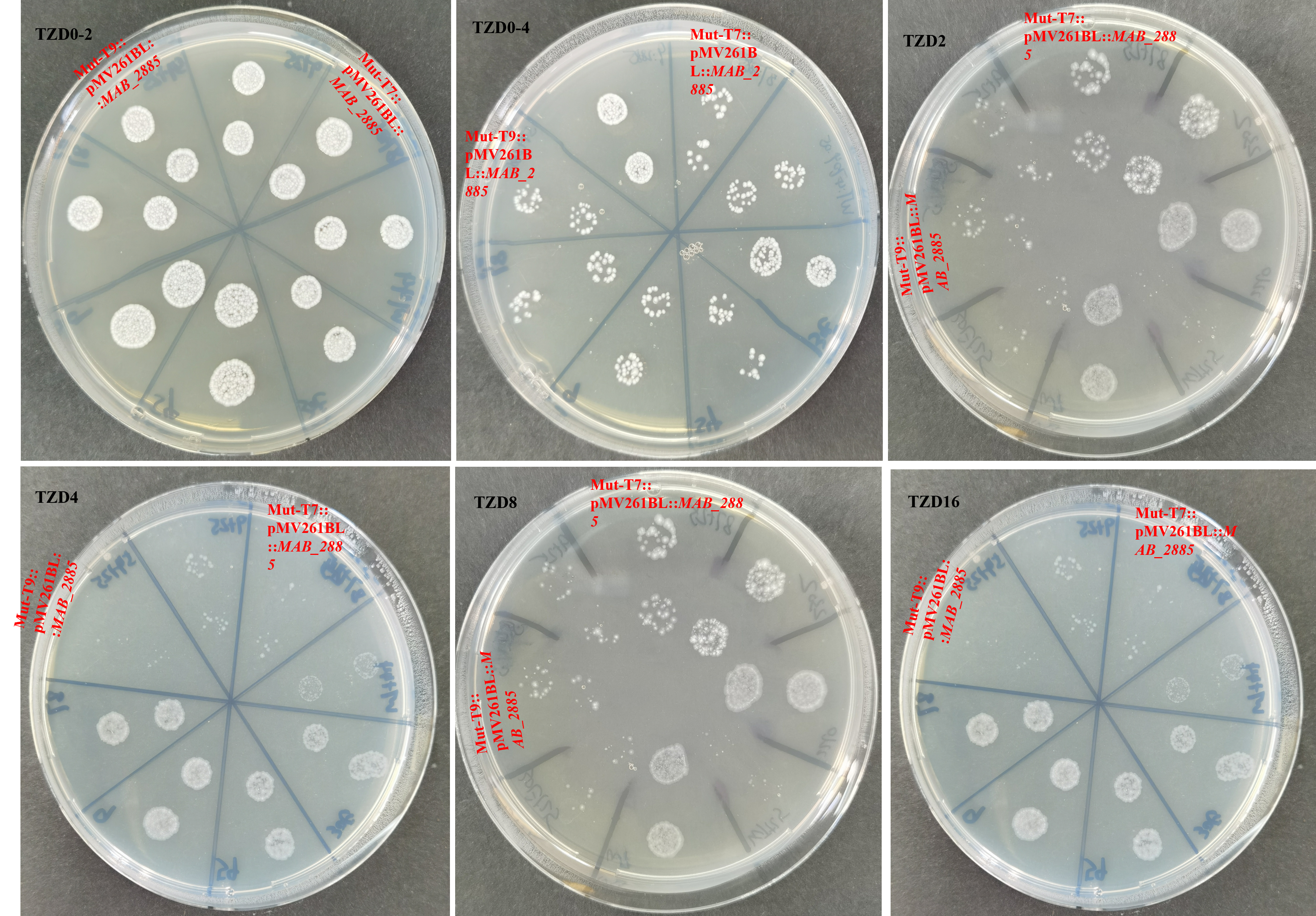

Supplement: S2 Data — (ZIP) [file ppat.1013190.s007.zip › S2_data/Complete, uncropped agar/Fig 1B-OEa.tif]

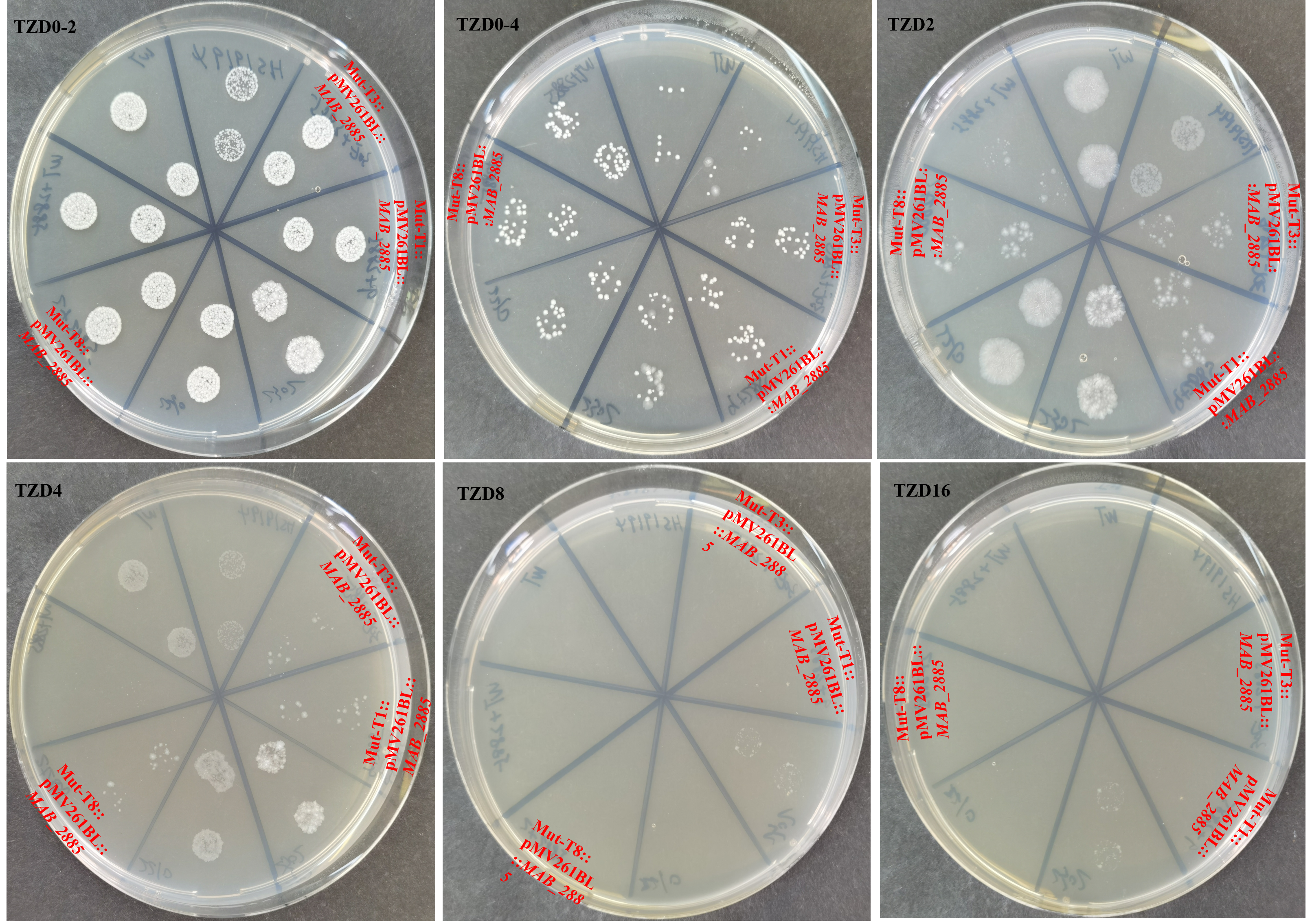

Supplement: S2 Data — (ZIP) [file ppat.1013190.s007.zip › S2_data/Complete, uncropped agar/Fig 1B-OEb.tif]

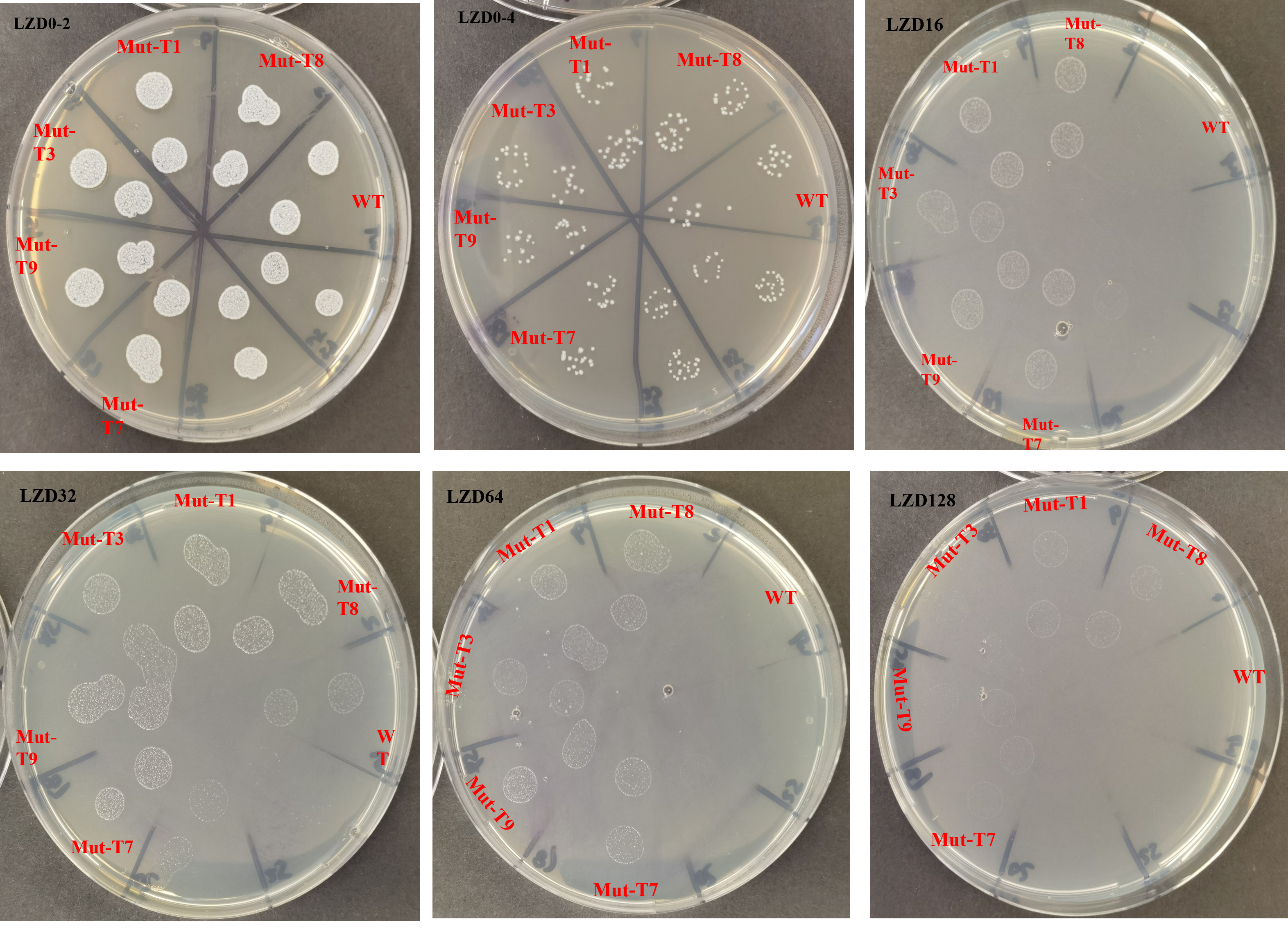

Supplement: S2 Data — (ZIP) [file ppat.1013190.s007.zip › S2_data/Complete, uncropped agar/Fig 1C.tif]

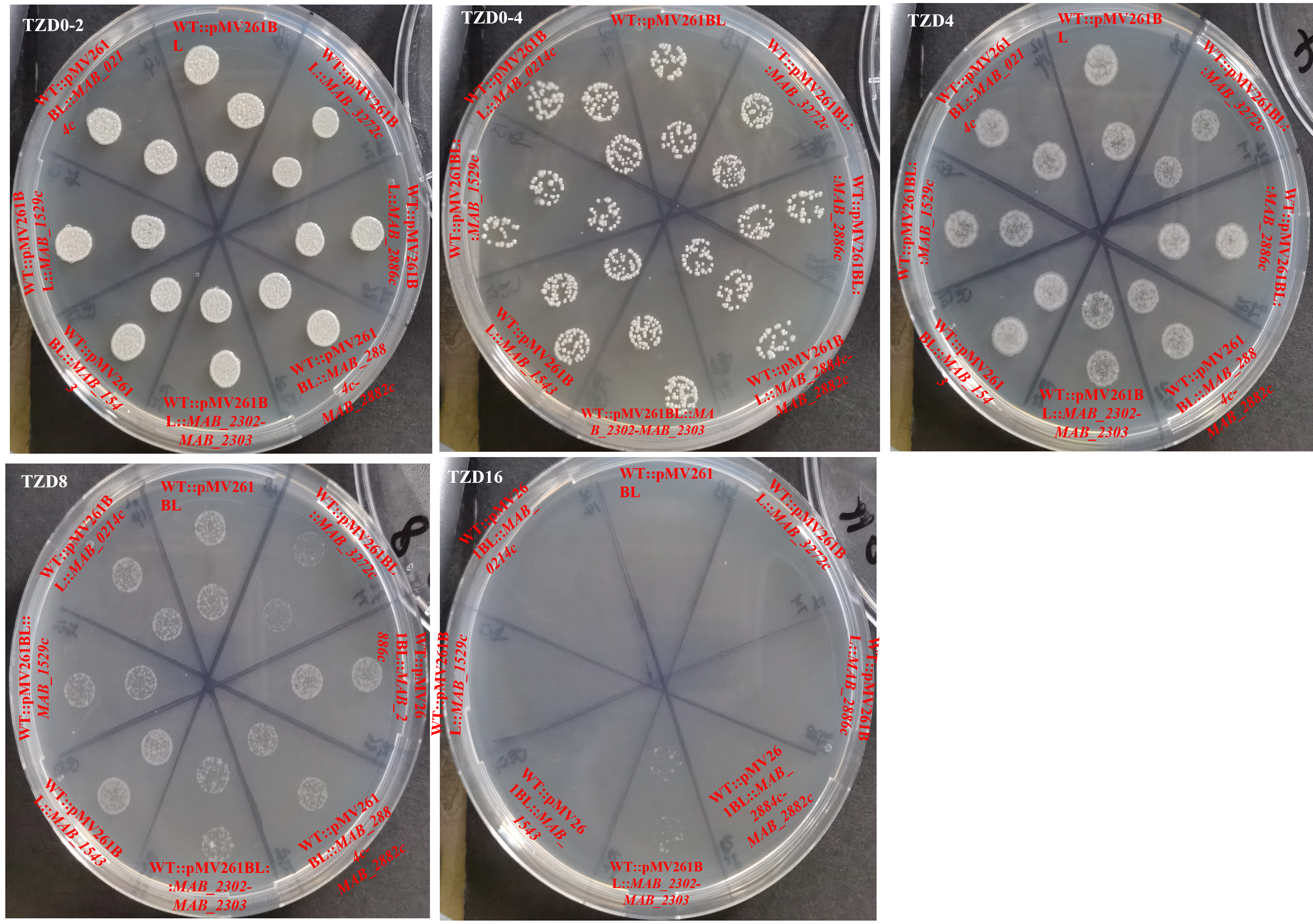

Supplement: S2 Data — (ZIP) [file ppat.1013190.s007.zip › S2_data/Complete, uncropped agar/Fig 2B.tif]

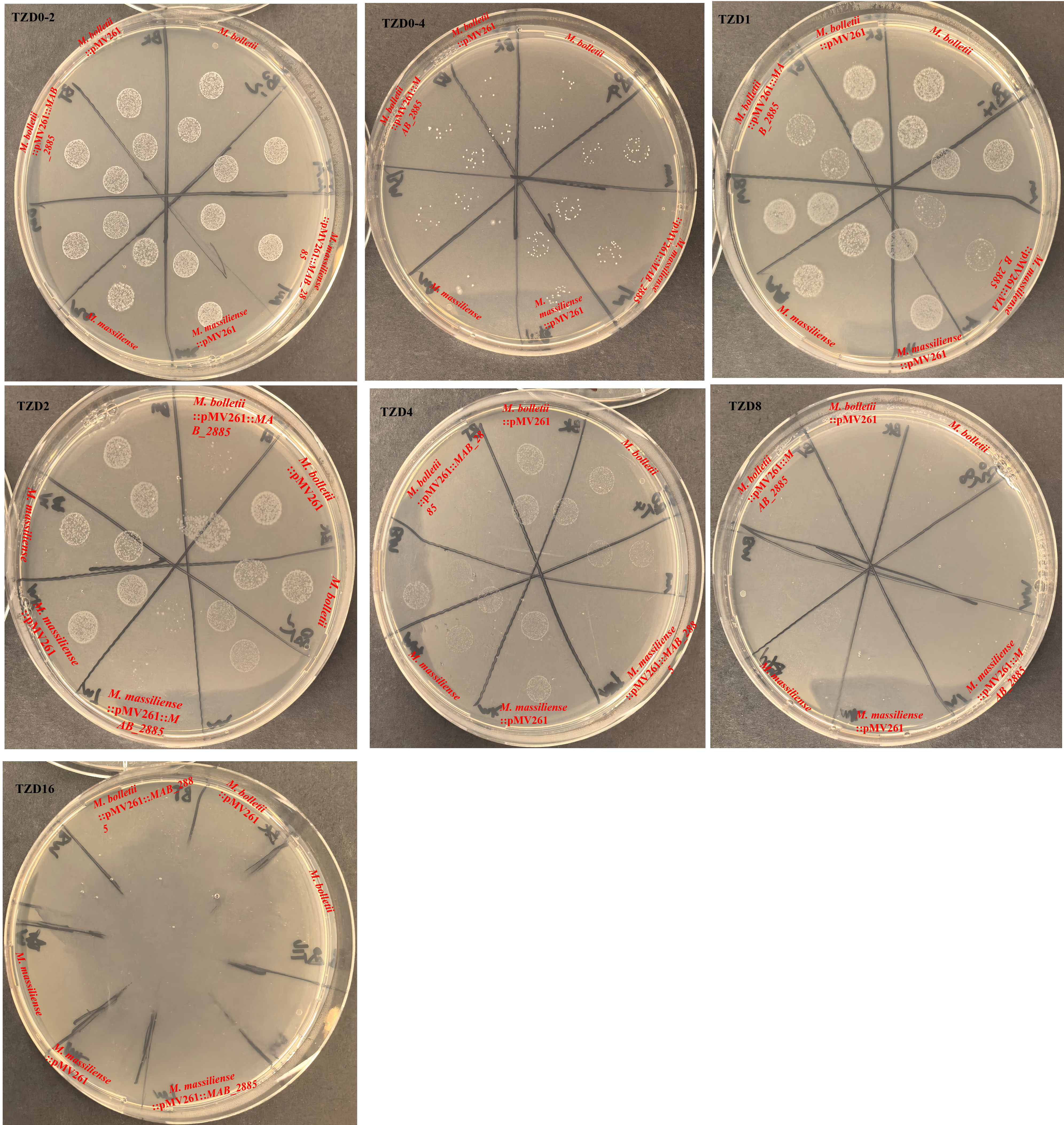

Supplement: S2 Data — (ZIP) [file ppat.1013190.s007.zip › S2_data/Complete, uncropped agar/Fig 5A.tif]

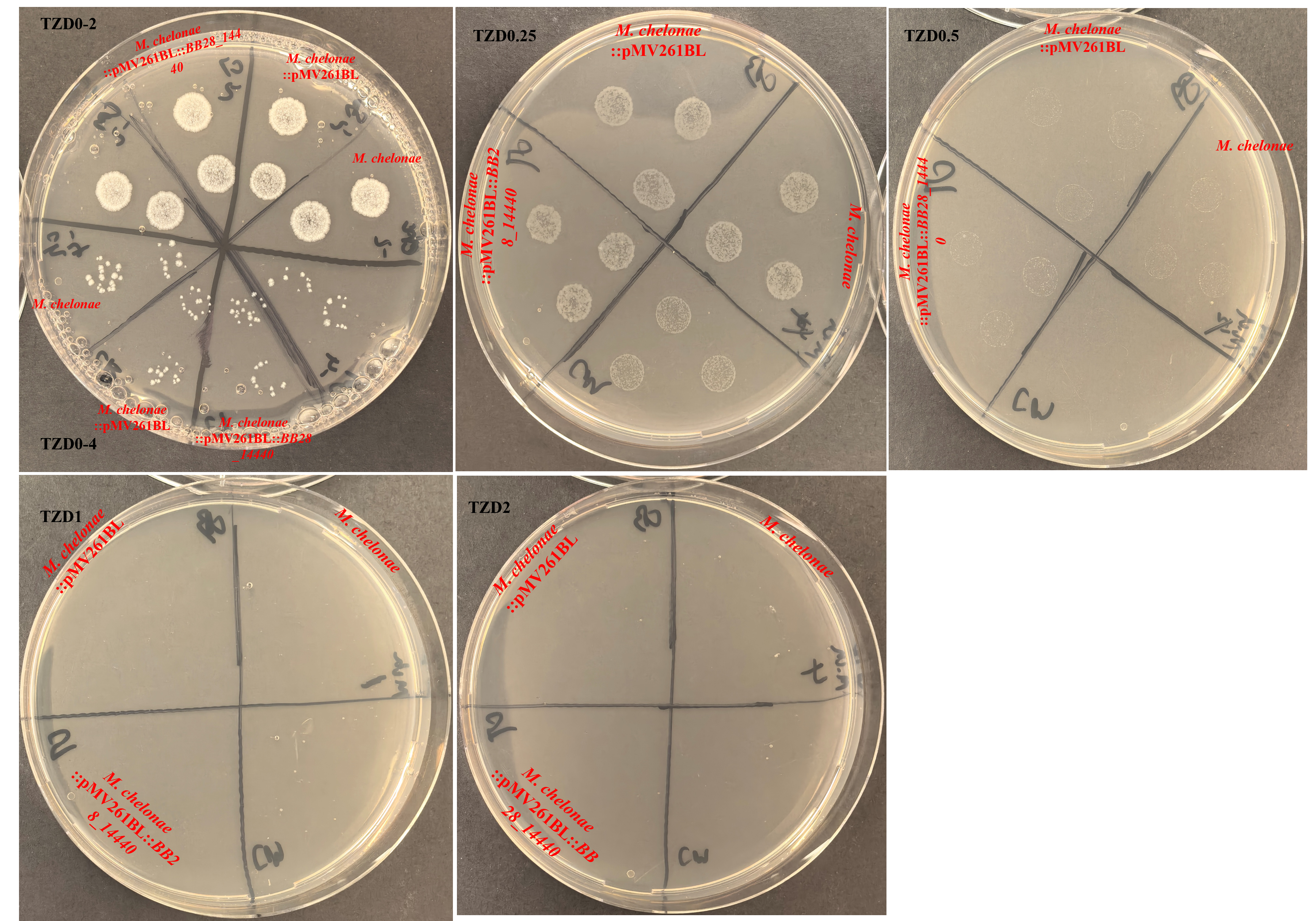

Supplement: S2 Data — (ZIP) [file ppat.1013190.s007.zip › S2_data/Complete, uncropped agar/Fig 5B.tif]

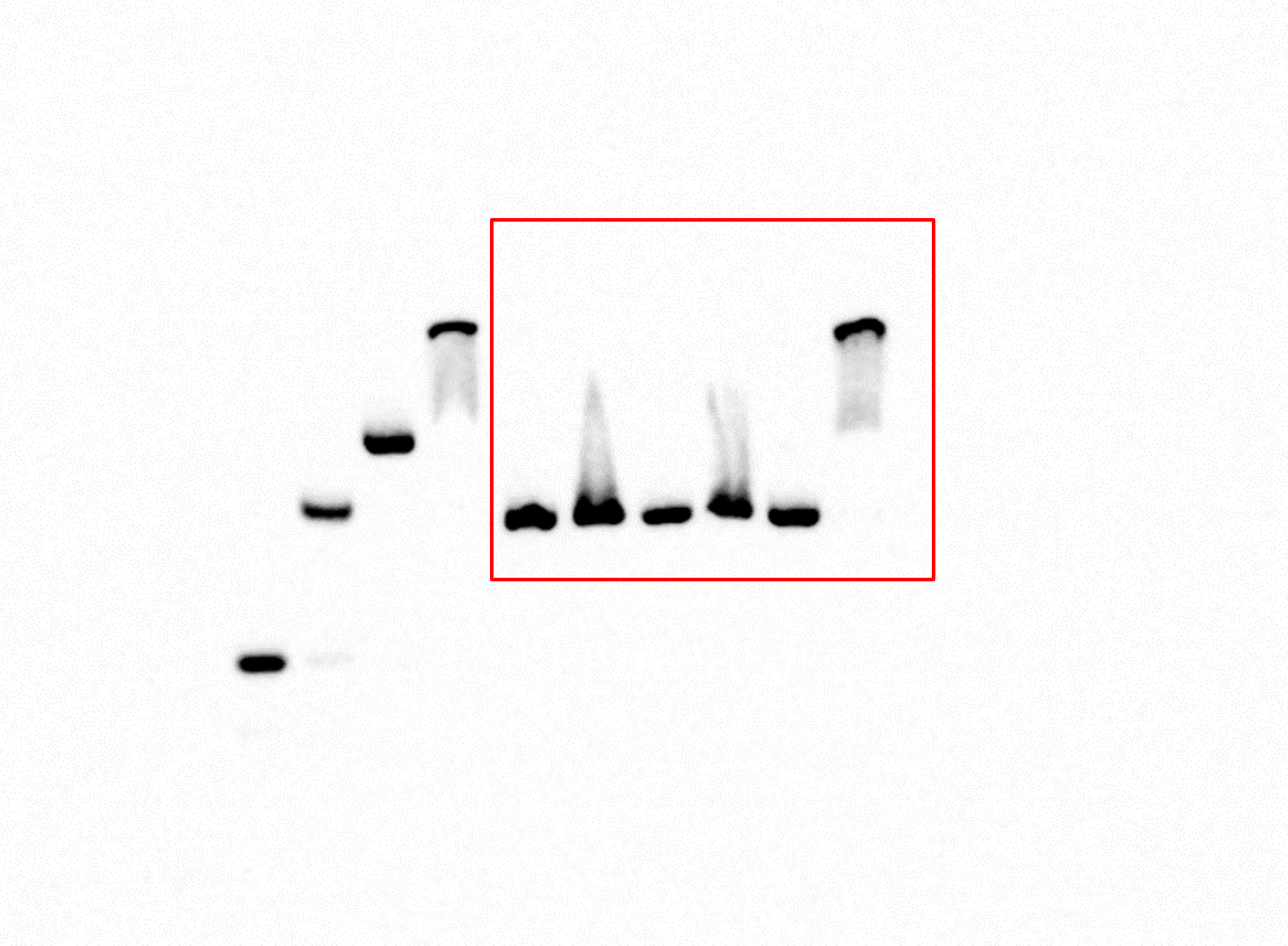

Supplement: S2 Data — (ZIP) [file ppat.1013190.s007.zip › S2_data/Complete, uncropped EMSA/Fig3A.tif]

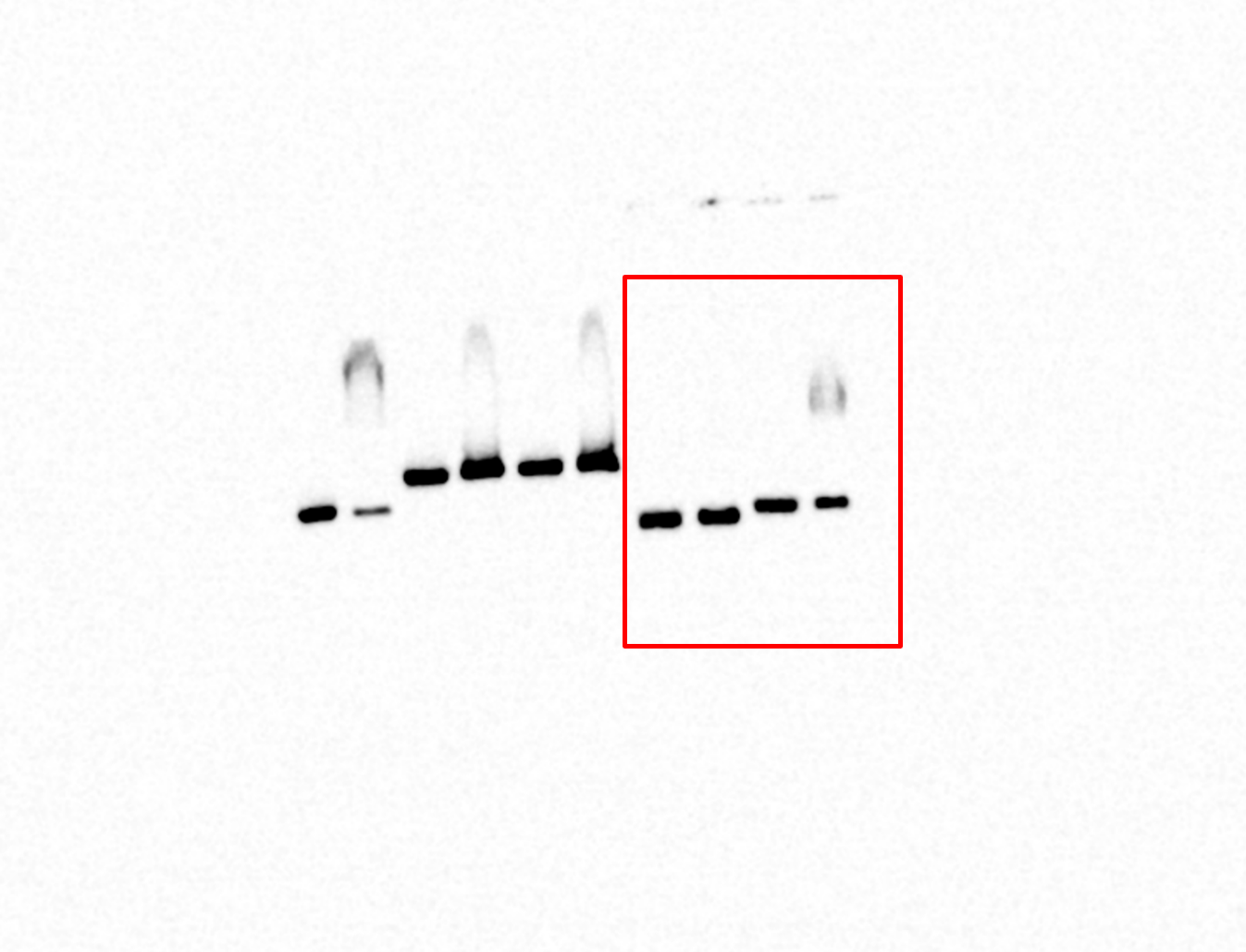

Supplement: S2 Data — (ZIP) [file ppat.1013190.s007.zip › S2_data/Complete, uncropped EMSA/Fig3B.tif]

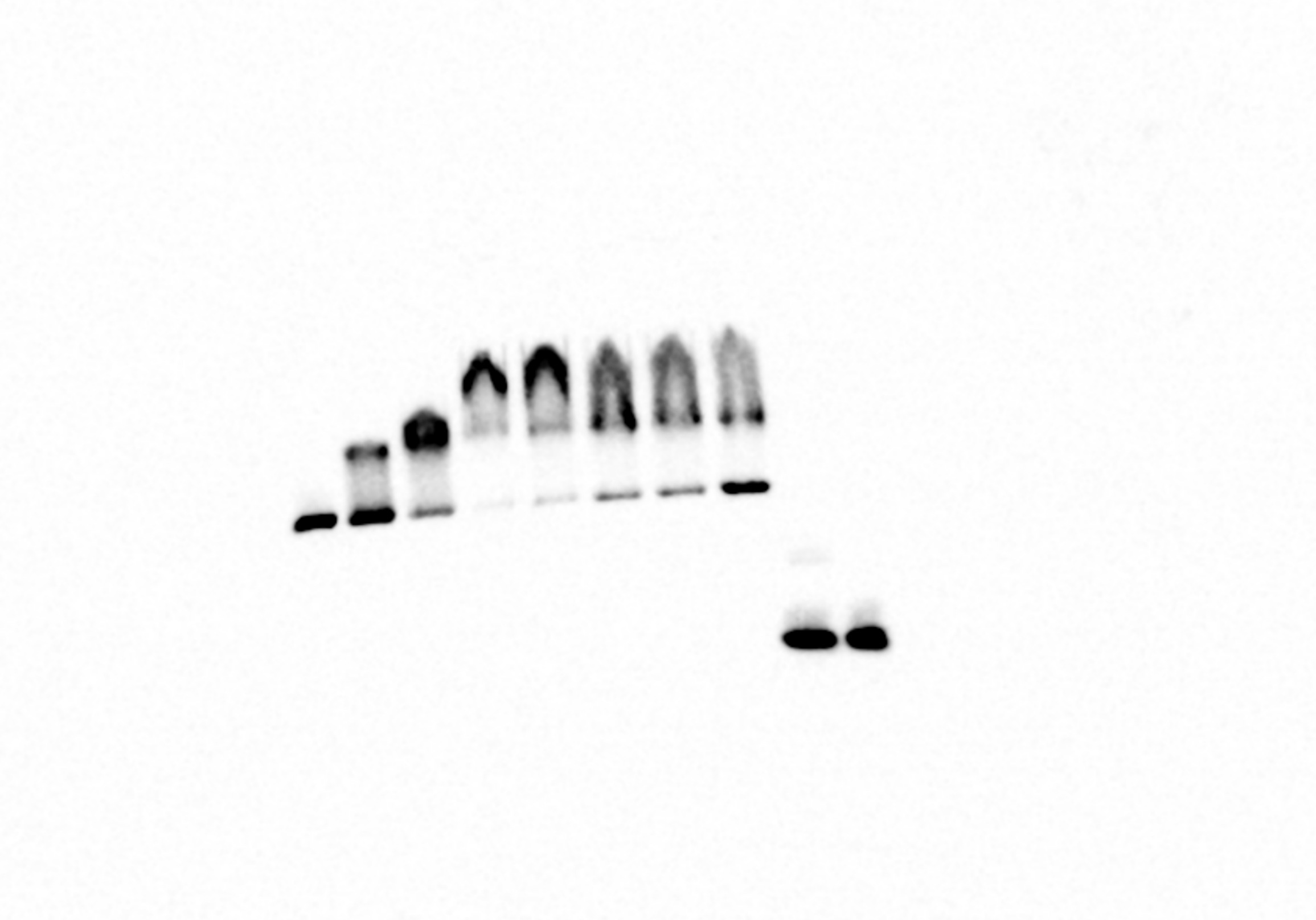

Supplement: S2 Data — (ZIP) [file ppat.1013190.s007.zip › S2_data/Complete, uncropped EMSA/Fig3C.tif]

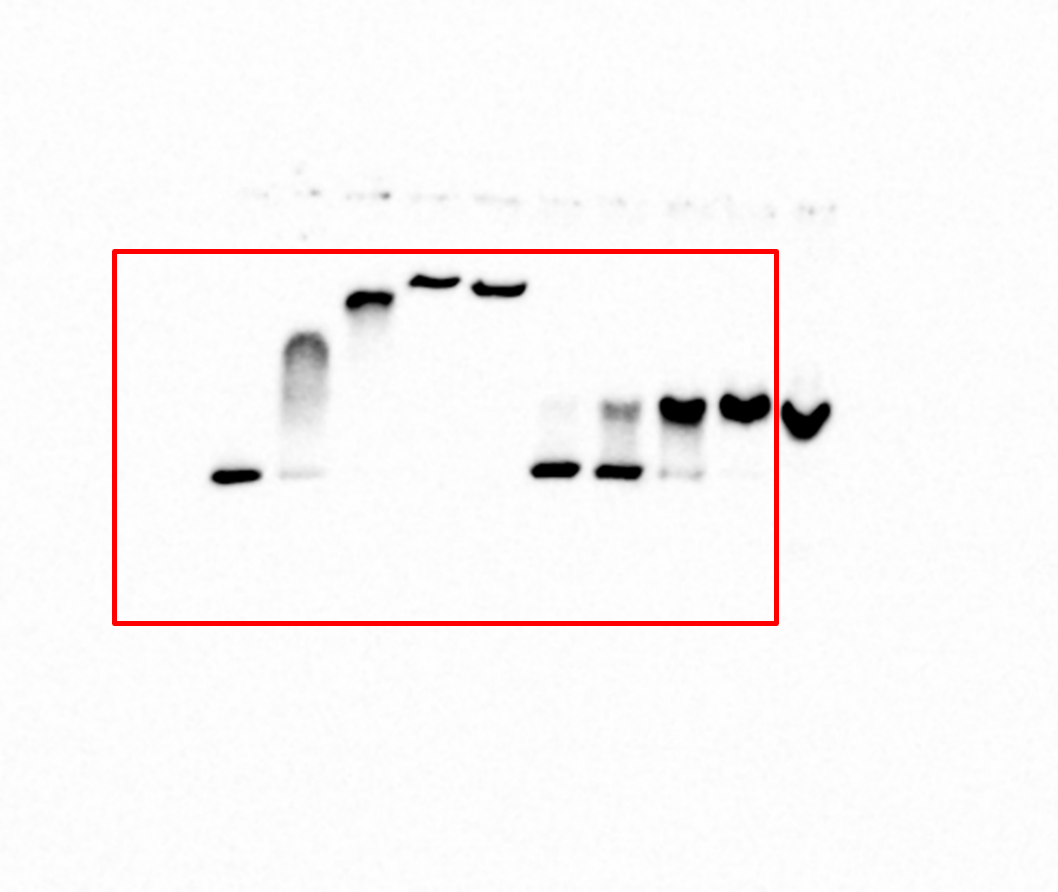

Supplement: S2 Data — (ZIP) [file ppat.1013190.s007.zip › S2_data/Complete, uncropped EMSA/Fig3D.tif]

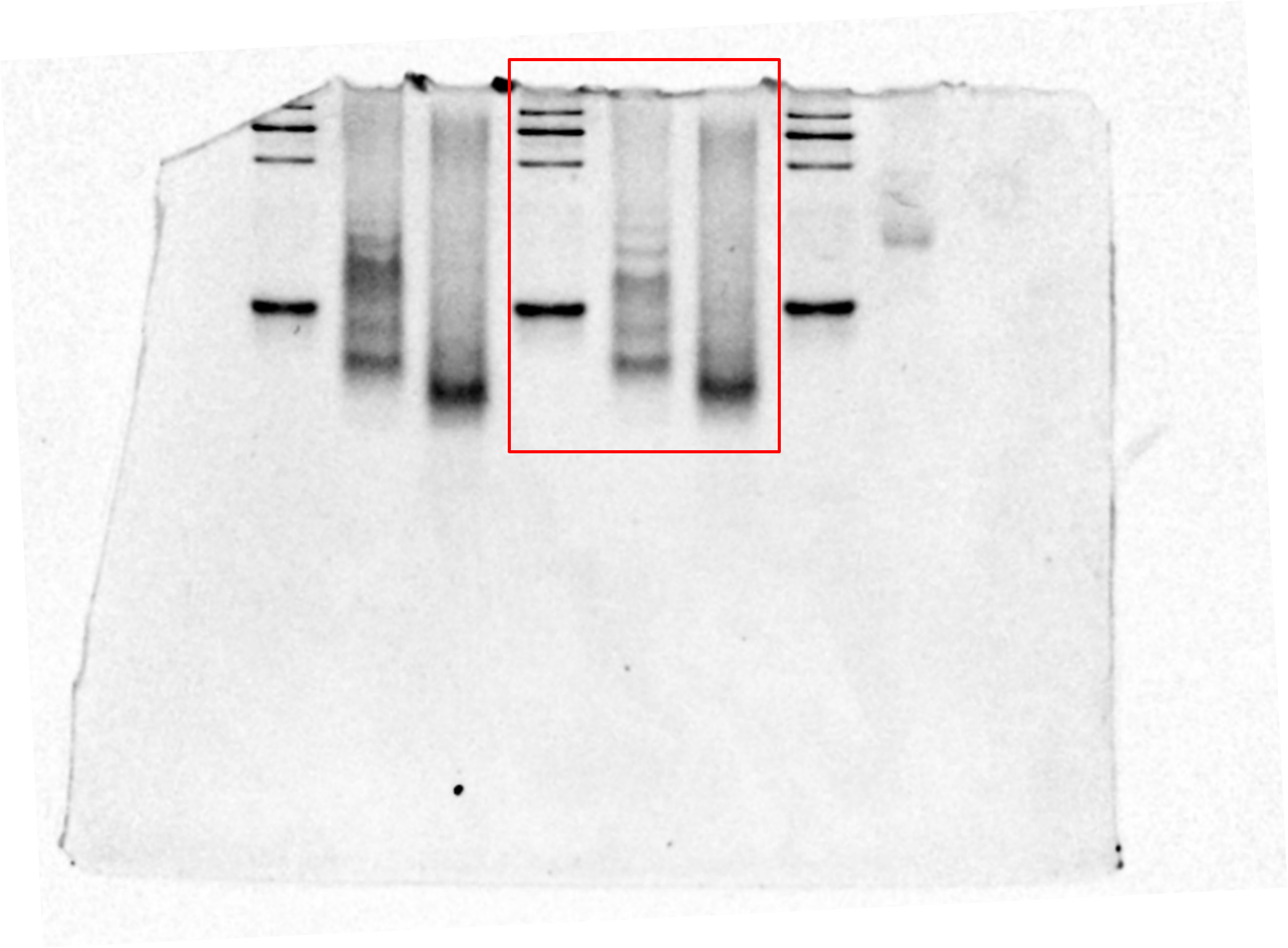

Supplement: S2 Data — (ZIP) [file ppat.1013190.s007.zip › S2_data/Complete, uncropped EMSA/Fig3E.tif]
